# A new species of the large-headed coastal marine turtle *Solnhofia* (Testudinata, Thalassochelydia) from the Late Jurassic of NW Switzerland

**DOI:** 10.7717/peerj.9931

**Published:** 2020-11-12

**Authors:** Jérémy Anquetin, Christian Püntener

**Affiliations:** 1 JURASSICA Museum, Porrentruy, Switzerland; 2 Department of Geosciences, University of Fribourg, Fribourg, Switzerland; 3 Naturmuseum Solothurn, Solothurn, Switzerland; 4 Section d’archéologie et paléontologie, Office de la culture, République et Canton du Jura, Porrentruy, Switzerland

**Keywords:** *Solnhofia*, Thalassochelydia, Testudinata, Kimmeridgian, Late Jurassic, Switzerland

## Abstract

**Background:**

The large-headed turtle *Solnhofia parsonsi* is known by a handful of specimens from the Late Jurassic of Germany and Switzerland (maybe also France). *Solnhofia parsonsi* is traditionally regarded as a “eurysternid” Thalassochelydia, a group of small to medium sized, mostly lagoonal or marginal turtles found almost exclusively in the Late Jurassic of Europe. More recently, *Solnhofia parsonsi* has been proposed to be a close relative of Sandownidae, an enigmatic group of Cretaceous to Paleogene turtles characterized by a derived cranial anatomy and a wider geographical distribution. Sandownids may therefore have evolved from thalassochelydian ancestors such as *Solnhofia parsonsi*.

**Methods:**

We herein describe new material of *Solnhofia* from the Kimmeridgian (Late Jurassic) of Porrentruy, NW Switzerland. The bulk of the material consists of an association of a cranium and over 180 shell bones found together in a block of marly limestone. A second cranium and a mandible from slightly younger, but nearby localities are also described.

**Results:**

We refer the new material to *Solnhofia brachyrhyncha* n. sp. The new species shares with *Solnhofia parsonsi* a relatively large head, an extensive secondary palate formed primarily by the maxillae, a greatly developed processus trochlearis oticum with a contribution from the parietal and quadratojugal, a large jugal-palatine contact in the floor of the fossa orbitalis, and a posteromedial process of the jugal running on the dorsal surface of the maxilla and pterygoid. Some of these characteristics are also present in sandownids, but our morphological study clearly shows that *Solnhofia brachyrhyncha* is closer to *Solnhofia parsonsi* than to any sandownids.

**Discussion:**

*Solnhofia brachyrhyncha* differs from *Solnhofia parsonsi* in many aspects, notably: a shortened and broader cranium, a shorter and posteriorly broader upper triturating surface with a slightly sinusoidal lateral margin and without contribution from the palatine, a processus trochlearis oticum more oblique in dorsal or ventral view and less concave in anterior view, choanae that do not extend posteriorly on the pterygoids, a more developed processus pterygoideus externus, a condylus mandibularis situated anterior to the level of the occipital plane, a greater ventral exposure of the parabasisphenoid, a mandible about as wide as long, a relatively short symphysis, a lower triturating surface widened posterolaterally thanks to the presence of large laterally projecting dentary tubercles, a stouter and shorter coronoid process, a splenial positioned more anteriorly along the mandibular ramus, costo-peripheral fontanelles extending more anteriorly and posteriorly along the costal series, and an escutcheon shaped central plastral fontanelle formed mostly by the hypoplastra. In addition to the morphology of the new species, we also briefly discuss about observable ontogenetic variations and possible taphonomic origin of the assemblage.

## Introduction

*Solnhofia parsonsi* is a remarkable Late Jurassic turtle characterized notably by a proportionally large head and the presence of a secondary palate formed primarily by the maxillae (Gaffney, 1975; Lapparent de Broin, Lange-Badré & Dutrieux, 1996; Joyce, 2000). This species was erected by Gaffney (1975) based on two skulls. The holotype (TM 4023), a specimen initially described but not named by Parsons & Williams (1961), consists of a cranium and associated mandible from an unknown locality (probably from the Solnhofen region). The second, referred specimen (NMS 8741, previously numbered NMS 137) is a deformed cranium originating from the Kimmeridgian of Solothurn, Switzerland. This specimen was briefly mentioned by Bräm (1965). Only a handful of specimens have since been referred to *Solnhofia*.

A juvenile specimen with a large head and roundish shell (MNHN CNJ 76) from the Tithonian lithographic limestones of Canjuers, France was initially attributed to aff. *Solnhofia* sp. (Fabre et al., 1982), but this identification was later questioned due to the poor quality of the preservation (Broin, 1994). Another specimen from Canjuers (MNHN CNJ 82) consisting of a cranium and associated scattered elements of the postcranium can be more confidently assigned to *Solnhofia* sp. based on the large head and elongation of the snout (Broin, 1994), but this specimen was never described in detail. Later, Lapparent de Broin, Lange-Badré & Dutrieux (1996) referred the posterior part of a cranium (ICHLU 005.4.20) from the Kimmeridgian of Labastide-Murat, France to *Solnhofia* aff. *parsonsi*. Finally, a well-preserved specimen (JM SCHA 70) from the Kimmeridgian/Tithonian boundary of Schamhaupten, Germany represents the first complete skeleton of *Solnhofia parsonsi* and documents the postcranial morphology of this taxon (Joyce, 2000).

*Solnhofia parsonsi* is usually considered to be a representative of Thalassochelydia, a clade of coastal marine turtles almost exclusively known from the Late Jurassic of western and central Europe (but at least one species reached Argentina, while at least another one survived into the Early Cretaceous) and that represents the first radiation of chelonians into marine environments (Anquetin, Püntener & Joyce, 2017; Anquetin & André, 2020; González Ruiz, De la Fuente & Fernández, 2020). Thalassochelydians are traditionally considered to be basal Pan-Cryptodira, but recent phylogenetic works have somewhat challenged this conclusion by recovering them as stem Testudines, sister group to Pleurodira, or stem Chelonioidea (Evers & Benson, 2019; Evers, Barrett & Benson, 2019; Gentry, Ebersole & Kiernan, 2019). These recent studies consistently found Thalassochelydia to form a clade with Sandownidae, a group of unique coastal marine turtles characterized by the presence of an extensive secondary palate and known from the Early Cretaceous to the Paleocene (Tong & Meylan, 2013). Possible relationships between sandownids and thalassochelydians, *Solnhofia parsonsi* in particular, were discussed several times in the literature (Joyce, 2007; Mateus et al., 2009; Evers & Benson, 2019). This hypothesis was further substantiated recently based on morphological arguments by Evers & Joyce (2020) who suggested that *Solnhofia parsonsi* could represent the earliest sandownid. However, these results have not yet been tested in a phylogenetic framework and the respective relationships of sandownids, thalassochelydians and *Solnhofia parsonsi* remain uncertain. For example, it is unclear whether sandownids and thalassochelydians are just sister clades and *Solnhofia* only belongs to one of them or if sandownids are included within thalassochelydians and derive from *Solnhofia*-like taxa. For the time being, we conservatively assign *Solnhofia* to Thalassochelydia herein.

In the present study, we report new material of *Solnhofia* from the Kimmeridgian of Porrentruy, Switzerland. The bulk of this new material consists of a dense assemblage containing a cranium and over 180 shell bones. This cranium and the best preserved shell bones are herein used to define the new taxon *Solnhofia brachyrhyncha* n. sp., which notably differs from the type species *Solnhofia parsonsi* in its shorter snout, posteriorly broad triturating surface, and escutcheon shaped central plastral fontanelle. A second cranium and an isolated mandible from nearby localities, but different stratigraphical layers are also confidently referred to *Solnhofia brachyrhyncha* n. sp.

## Materials and Methods

### Material

**Figure 1:**
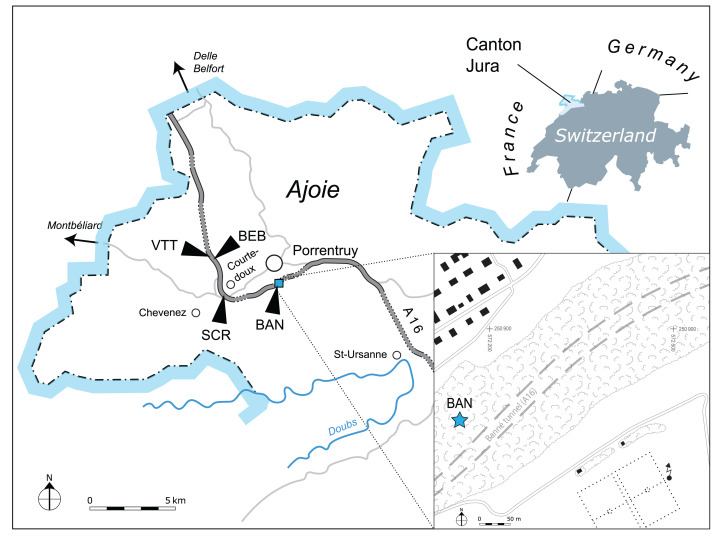
Geographical map of the Ajoie region, Canton of Jura, Switzerland. The position of the different localities mentioned in the present study is indicated along the A16 Highway (see text for abbreviations). Inset: the Banné (BAN) site is located in a forest on the Banné hill, southwest of Porrentruy.

The new material presented herein was unearthed during the construction of the A16 Transjurane highway in NW Switzerland. Thanks to generous financial support from the Swiss Confederation, the archeological and paleontological material discovered during this construction was systematically collected and documented. In recent years, several studies focussing on the rich Kimmeridgian strata in the region of Porrentruy (Fig. 1) were published, including articles on plant remains (Philippe et al., 2010), invertebrates (Schudack et al., 2013; Koppka, 2015), fishes (Leuzinger et al., 2015, 2017; Leuzinger, Püntener & Billon-Bruyat, 2017; Leuzinger et al., 2020), dinosaur tracks (Razzolini et al., 2017; Marty et al., 2018; Castanera et al., 2018), and turtles (Püntener et al., 2014; Anquetin, Püntener & Billon-Bruyat, 2015; Püntener, Anquetin & Billon-Bruyat, 2015, 2017a, 2020).

MJSN BAN001-2 originally consisted of a large block of marly limestone which was found entangled in the roots of a deracinated tree in 2001 on the northern slope of the Banné locality (BAN; 47 24′ 23.52″N 7 4′ 20.35″E). “Le Banné” corresponds to a wooded hill that borders the southwest residential area in Porrentruy. Under this hill runs the Banné tunnel of the A16 highway (Fig. 1). The block contained an assemblage of numerous turtle shell bones, which were at the time interpreted as remains of juveniles because of their relatively small size. Some of these shell bones were initially prepared and removed from the matrix, but many were simply surface prepared and left in the block of matrix. Several years later, the identification of a turtle cranium among the remains still embedded in the matrix prompted the preparation of the complete block. MJSN BAN001-2 now consists of a collection of over 180 disarticulated shell bones and a cranium. A selection of the best preserved remains were given a specific second level identification number for the purpose of this study (Table 1). MJSN BAN001-2.1 (the cranium) and MJSN BAN001-2.2 to MJSN BAN001-2.28 (shell remains) are referred to the new species *Solnhofia brachyrhyncha*. A pair of hypoplastra (MJSN BAN001-2.29) is tentatively assigned to a juvenile specimen of *Tropidemys*? *langii*? (see below). All other bones from this assemblage are referred to Thalassochelydia indet.

**Table 1:** Identified and figured elements of the assemblage MJSN BAN001-2. This table summarizes the bones of the assemblage MJSN BAN001-2 that were given second level identification numbers (format MJSN BAN001-2.x). For each element, we provide an identification and a reference to the figure in which the element is illustrated.

Specimen number	Identification	Figures
MJSN BAN001-2.1	Skull	3, 4A and 4B
MJSN BAN001-2.2	Costal 1 (left)	8A
MJSN BAN001-2.3	Costal 1 (left)	8B
MJSN BAN001-2.4	Costal 1 (right)	8C
MJSN BAN001-2.5	Costal 1 (right)	8D
MJSN BAN001-2.6	Costal 1 (right)	8E
MJSN BAN001-2.7	Costal 1 (right)	8F
MJSN BAN001-2.8	Costal 5 (left)	10A
MJSN BAN001-2.9	Costal 5 (left)	10B
MJSN BAN001-2.10	Costal 5 (left)	10C
MJSN BAN001-2.11	Costal 5 (right)	10D
MJSN BAN001-2.12	Costal 5 (right)	10E
MJSN BAN001-2.13	Costal 5 (right)	10F
MJSN BAN001-2.14	Peripheral (bridge)	12A–12C
MJSN BAN001-2.15	Peripheral (bridge)	12D–12F
MJSN BAN001-2.16	Peripheral (bridge)	12G–12I
MJSN BAN001-2.17	Peripheral (bridge)	12J–12L
MJSN BAN001-2.18	Hyoplastron (right)	13A
MJSN BAN001-2.19	Hyoplastron (right)	13B
MJSN BAN001-2.20	Hyoplastron (left)	13C
MJSN BAN001-2.21	Hyoplastron (left)	13D
MJSN BAN001-2.22	Hyoplastron (left)	13E
MJSN BAN001-2.23	Hyoplastron (left)	13F
MJSN BAN001-2.24	Hyoplastron (left)	13G
MJSN BAN001-2.25	Hypoplastron (right)	14A
MJSN BAN001-2.26	Hypoplastron (right)	14B
MJSN BAN001-2.27	Hypoplastron (left)	14C
MJSN BAN001-2.28	Hypoplastron (left)	14D
MJSN BAN001-2.29	Hypoplastra *Tropidemys*? *langii*?	16

MJSN SCR010-1214 consists of a severely crushed turtle skull lacking most of the ventral and anterior parts. MJSN BEB011-13 is a relatively small turtle mandible that was found in a dinosaur track infilling (see Geological settings). This is one of the rare vertebrate remains found in the dinosaur-track-bearing layers of the Porrentruy region (Püntener, Anquetin & Billon-Bruyat, 2017a; Püntener et al., 2019).

### Geological settings

**Figure 2:**
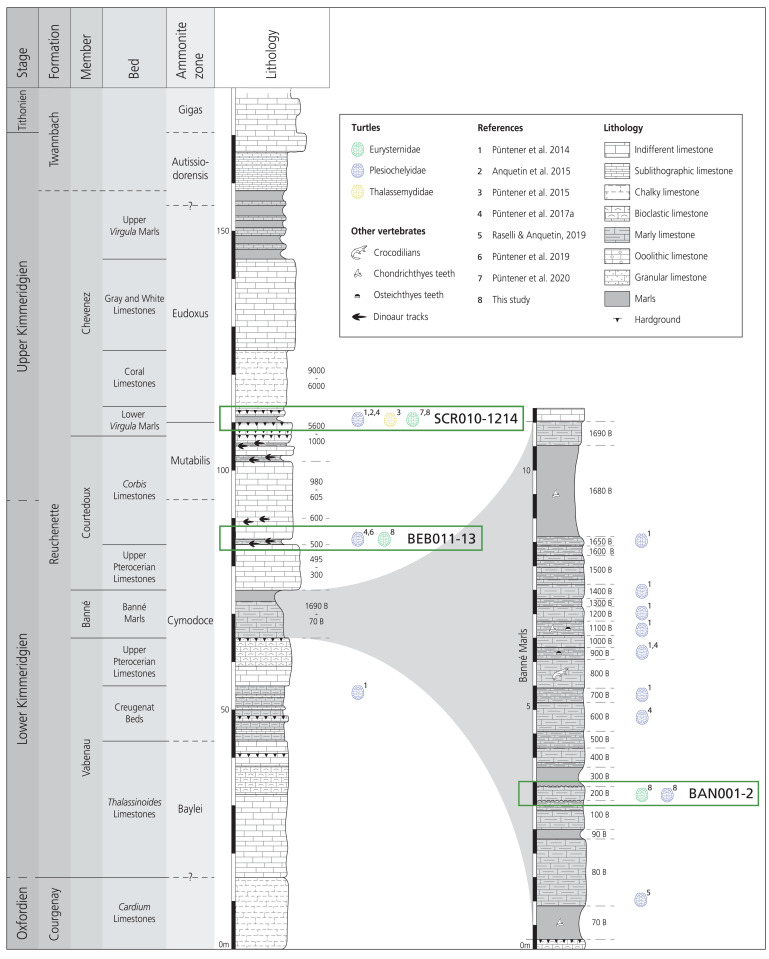
Stratigraphic section of the Reuchenette Formation in Ajoie, Canton of Jura, Switzerland, with a close-up on the Banné Marls. The specimens described in this study are indicated by their collection number and circled in green. Main published findings of “Eurysternidae” (green), “Thalassemydidae” (yellow) and “Plesiochelyidae” (blue) in the area are figured by colored turtle shell icons. Corresponding literature is indicated. Scheme modified after Comment et al. (2015) and Raselli & Anquetin (2019).

The Mesozoic sediments of the Porrentruy region belong to the tabular portion of the Jura Mountains (Marty et al., 2007). During the Kimmeridgian, this region was part of a SW-NE trending carbonate platform with various depositional environments such as lagoons, channels, and the littoral zone (Marty & Hug, 2003; Colombié & Strasser, 2005; Marty, 2008). These rapidly changing sedimentation systems led to the formation of the fossil-rich layers of the Banné Marls, the Courtedoux Member, and the Lower *Virgula* Marls (Marty & Hug, 2003; Comment et al., 2015; Fig. 2).

The assemblage MJSN BAN001-2 was extracted from the Banné Marls in the Banné hill (SW of Porrentruy; Fig. 1). The approximately 10-m thick Banné Marls (early Kimmeridgian, Reuchenette Formation, Banné Member, Cymodoce Ammonite Zone; Fig. 2; Comment et al., 2015) consist of “grey, dm-thick layers of marlstones, calcarenitic marls, and marly limestones” (Jank, Wetzel & Meyer, 2006). They are especially rich in invertebrates (Koppka, 2015), but also contain remains of vertebrates such as turtles (Püntener et al., 2014; Püntener, Anquetin & Billon-Bruyat, 2017a, 2017b), crocodilians (Schaefer, Püntener & Billon-Bruyat, 2018), and fishes (Leuzinger et al., 2017; Leuzinger, Püntener & Billon-Bruyat, 2017). The turtle fauna of the Banné Marls is dominated by the “plesiochelyids” *Tropidemys langii* and (less frequent) *Plesiochelys bigleri* (Püntener et al., 2014; Püntener, Anquetin & Billon-Bruyat, 2017a; Raselli & Anquetin, 2019). However, most of the turtle material of the Banné Marls does not originate from the Banné (BAN) site, but comes from stratigraphically younger layers excavated at the Vâ Tche Tchâ (VTT) site, which is situated about four kilometers northwest of the Banné (BAN) site (Figs 1 and 2).

The poorly preserved cranium MJSN SCR010-1214 originates from the Lower *Virgula* Marls (late Kimmeridgian, Reuchenette Formation, Chevenez Member, Eudoxus Ammonite Zone; Fig. 2; Comment et al., 2015). This level is rich in vertebrate and wood remains (Philippe et al., 2010; Leuzinger et al., 2017; Leuzinger, Püntener & Billon-Bruyat, 2017; Püntener, Anquetin & Billon-Bruyat, 2017b; Schaefer, Püntener & Billon-Bruyat, 2018). The better part of the turtle remains from the Porrentruy region comes from the Lower *Virgula* Marls (Anquetin, Püntener & Billon-Bruyat, 2015; Püntener, Anquetin & Billon-Bruyat, 2015, 2017a, 2020).

The mandible MJSN BEB011-13 was found in layer 501 at the base of the *Corbis* Limestones (early Kimmeridgian, Reuchenette Formation, Courtedoux Member, Cymodoce Ammonite Zone; Fig. 2; Comment et al., 2015). The Courtedoux Member is situated between the Banné Marls and the Lower *Virgula* Marls. This member is especially renowned for its dinosaur footprint bearing layers (Marty & Hug, 2003; Marty et al., 2007, 2018; Razzolini et al., 2017).

### Anatomical comparisons and 3D models

The new material described herein was primarily compared to the available material of *Solnhofia parsonsi*. For the cranium and mandible, the three main specimens we used for comparison were TM 4023 (holotype of *Solnhofia parsonsi*), NMS 8741 and JM SCHA 70. The mandible is only known in TM 4023 and JM SCHA 70. For TM 4023, we used the 3D model of Evers & Benson (2018) as primary source of data, completed by the available literature (Parsons & Williams, 1961; Gaffney, 1975; Evers & Joyce, 2020). We had NMS 8741 at hand during this study. Joyce (2000) was used for JM SCHA 70. In the following description, we refer to the relevant specimens when comparing *Solnhofia brachyrhyncha* to *Solnhofia parsonsi*. On rare occasions, we also used the specimens MNHN CNJ 82 and ICHLU 005.4.20 (see Introduction) based on personal observations. Following the work of Evers & Joyce (2020), the new material was also compared with sandownids when appropriate. These comparisons were made based on the published literature (Mateus et al., 2009; Tong & Meylan, 2013; Cadena, 2015; Evers & Joyce, 2020).

The 3D model of the cranium and mandible TM 4023 (*Solnhofia parsonsi*) was kindly provided by Evers & Benson (2018) and is available on Morphosource (see Additional Information). A textured 3D surface model of the cranium MJSN BAN001-2.1 (herein designated as the holotype of the new species *Solnhofia brachyrhyncha*) was also produced by photogrammetry (Anquetin & Püntener, 2020). This model can be downloaded from the MorphoMuseuM platform (see Additional Information).

For the shell, JM SCHA 70 is the only known specimen referred to *Solnhofia parsonsi* in which the shell is sufficiently known. For this specimen, we used mainly the original description of Joyce (2000). Concerning the plastral elements herein referred to *Tropidemys*? *langii*?, we used comparative material from different Kimmeridgian localities in the region of Porrentruy, Switzerland, as described previously by one of us (Püntener et al., 2014). When needed, comparisons were extended to other thalassochelydians, notably material from Solothurn and Porrentruy following some of our recent works (Anquetin, Püntener & Billon-Bruyat, 2014; Püntener, Anquetin & Billon-Bruyat, 2017a).

### Nomenclatural act

The electronic version of this article in Portable Document Format (PDF) will represent a published work according to the International Commission on Zoological Nomenclature (ICZN), and hence the new names contained in the electronic version are effectively published under that Code from the electronic edition alone. This published work and the nomenclatural acts it contains have been registered in ZooBank, the online registration system for the ICZN. The ZooBank LSIDs (Life Science Identifiers) can be resolved and the associated information viewed through any standard web browser by appending the LSID to the prefix http://zoobank.org/. The LSID for this publication is: [urn:lsid:zoobank.org:pub:C999A877-3080-490B-8202-6E98B621366E]. The online version of this work is archived and available from the following digital repositories: PeerJ, PubMed Central and CLOCKSS.

## Description of *Solnhofia brachyrhyncha* n. sp.

### Cranium

#### General description

**Figure 3:**
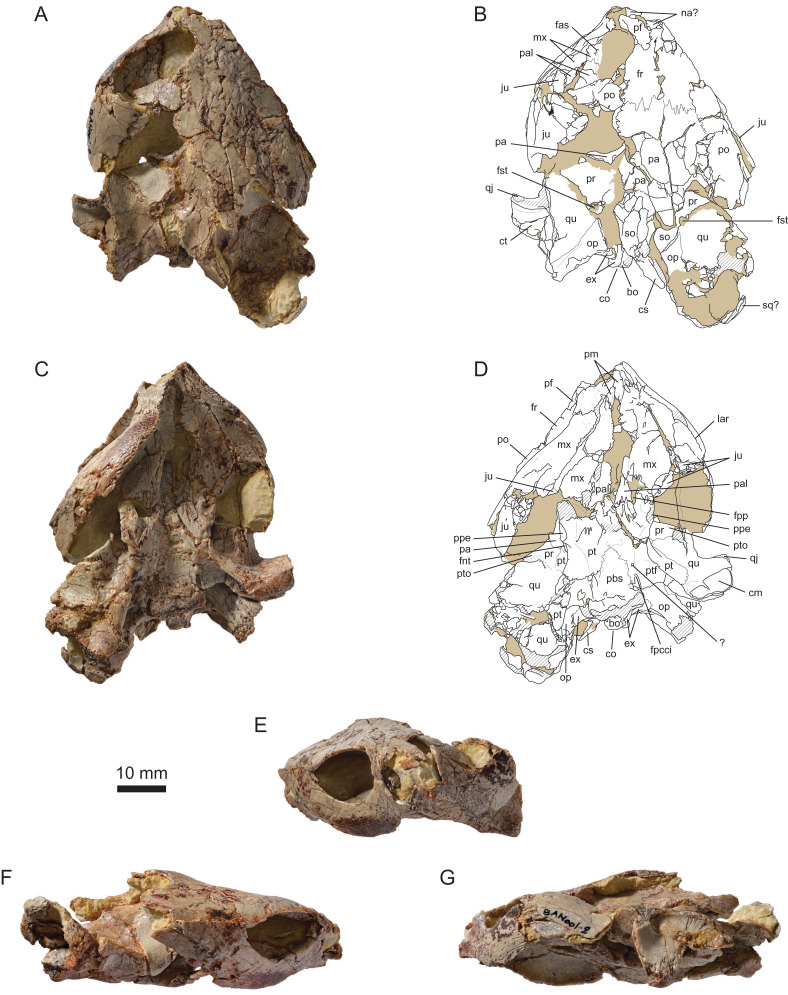
MJSN BAN001-2.1, holotype of *Solnhofia brachyrhyncha* (Kimmeridgian, Porrentruy, Switzerland). (A) Cranium in dorsal view; (B) interpretative drawing of the cranium in dorsal view; (C) cranium in ventral view; (D) interpretative drawing of the cranium in ventral view; (E) cranium in anterior view; (F) cranium in right lateral view; (G) cranium in left lateral view. In the drawings, hatchings correspond to damaged areas, while the light brown color represents the remaining matrix. Abbreviations: bo, basioccipital; cm, condylus mandibularis; co, condylus occipitalis; cs, crista supraoccipitalis; ct, cavum tympani; ex, exoccipital; fas, foramen alveolare superius; fnt, foramen nervi trigemini; fpcci, foramen posterius canalis carotici interni; fpp, foramen palatinum posterius; fr, fontal; fst, foramen stapedio-temporale; ju, jugal; lar, labial ridge; mx, maxilla; na, nasal; op, opisthotic; pa, parietal; pal, palatine; pbs, parabasisphenoid; pf, prefrontal; pm, premaxilla; po, postorbital; ppe, processus pterygoideus externus; pr, prootic; pt, pterygoid; ptf, pterygoid fossa; pto, processus trochlearis oticum; qj, quadratojugal; qu, quadrate; so, supraoccipital; sq, squamosal; ?, unnamed foramen.

**Figure 4:**
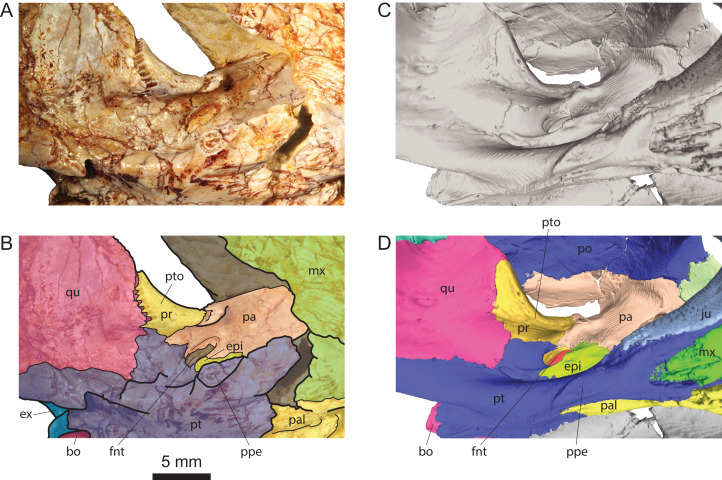
Comparison of the region of the processus trochlearis oticum and foramen nervi trigemini in *Solnhofia brachyrhyncha* and *Solnhofia parsonsi*. (A) Photograph of the cranium MJSN BAN001-2.1 (*So. brachyrhyncha*) in right anteroventral view; (B) same as in (A) with bones and sutures highlighted; (C) three-dimensional rendering of the cranium TM 4023 (*So. parsonsi*) in right anteroventral view; (D) same as in (C) with bones highlighted. (C) and (D) from Evers & Benson (2018). Abbreviations: bo, basioccipital; epi, epipterygoid; ex, exoccipital; fnt, foramen nervi trigemini; mx, maxilla; pa, parietal; pal, palatine; ppe, processus pterygoideus externus; pr, prootic; pt, pterygoid; pto, processus trochlearis oticum; qu, quadrate.

The cranium MJSN BAN001-2.1 is relatively complete, but has suffered an intense postmortem deformation (Figs. 3 and 4). The skull was compressed obliquely from the anterodorsal left side toward the posteroventral right side, causing much of the right side of the skull to collapse ventrolaterally and posteriorly. Most of the bones are crisscrossed by numerous cracks that make many sutures difficult to see. The tip of the snout and the area around the apertura narium externa, most of the upper and lower temporal regions, and the squamosals are missing. Disarticulation occurred in several places, notably between the left otic chamber and the roof of the cavum cranii, along the left postorbital region, and over much of the right palatoquadrate area and right otic chamber (Figs. 3A–3D). Postmortem deformation and disarticulation must be taken into account when interpreting the morphology of this specimen. The left side of the cranium is generally the one that is closer to the natural condition. No scute sulcus is preserved on the skull roof.

The general impression is that the snout of MJSN BAN001-2.1 is not as elongated as the one of TM 4023, NMS 8741 or JM SCHA 70. The triturating surface also appears to be broader posteriorly (Figs. 3C and 3D; see Maxilla below). These differences in cranial shapes are reflected in the general dimensions of the available specimens (Table 1). Skulls referred to *Solnhofia parsonsi* are longer than wide (length/width ratio ranging approximatively from 1.30 to 1.45), while the skull of *Solnhofia brachyrhyncha* is only slightly longer than wide (length/width ration of about 1.10). Since most available specimens are either incomplete or deformed somehow, these measurements and proportions must be handled with care, but the general trend remains true. *Solnhofia parsonsi* (JM SCHA 70) is characterized by a large head representing 40% of the carapace length (Joyce, 2000). Unfortunately, the cranium MJSN BAN001-2.1 is not associated with particular shell remains so its relative size cannot be determined with precision. However, if the estimated size range of the specimens represented in the assemblage MJSN BAN001-2 (150–250 mm in carapace length) is taken into account, we can surmise that *Solnhofia brachyrhyncha* is also a large-headed species with a skull representing between 25.7% and 42.8% of the carapace length.

**Figure 5:**
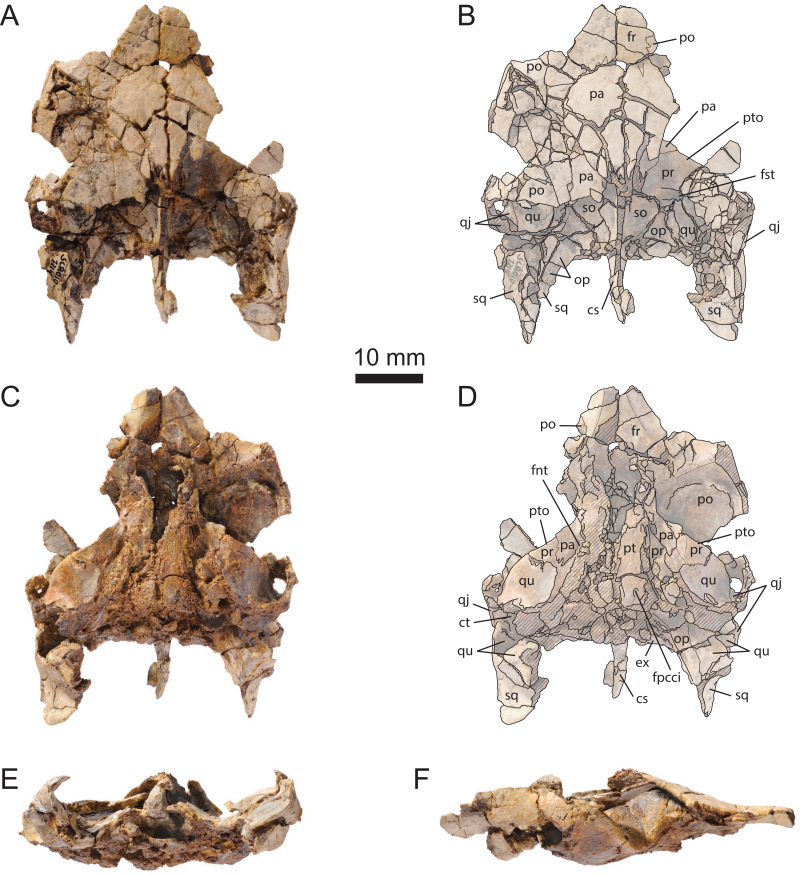
MJSN SCR010-1214, cranium of *Solnhofia brachyrhyncha* (Kimmeridgian, Porrentruy, Switzerland). (A) cranium in dorsal view; (B) interpretative drawing of the cranium in dorsal view; (C) cranium in ventral view; (D) interpretative drawing of the cranium in ventral view; (E) cranium in posterior view; (F) cranium in right lateral view. Note that the ventral aspect of the basicranium is severely eroded, while the palatal and orbital regions are missing. Hatchings correspond to damaged areas. Abbreviations: cs, crista supraoccipitalis; ct, cavum tympani; ex, exoccipital; fnt, foramen nervi trigemini; fr, frontal; fpcci, foramen posterius canalis carotici interni; fst, foramen stapedio-temporale; op, opisthotic; pa, parietal; po, postorbital; pr, prootic; pt, pterygoid; pto, processus trochlearis oticum; qj, quadratojugal; qu, quadrate; so, supraoccipital; sq, squamosal.

The preservation state of the cranium MJSN SCR010-1214 is relatively poor (Fig. 5). The cranium is severely crushed dorsoventrally (Figs. 5E and 5F) and most of its ventral aspect is eroded (Figs. 5C and 5D). The palatal, maxillary, nasal, and most of the orbital regions are missing. Most of the preserved bones are densely fractured, except those forming the dorsal part of the otic chambers. Despite its poor preservation state, this skull documents areas that are badly preserved or absent in the holotype cranium, as highlighted in the following detailed description.

#### Nasal

Nasals are well developed in most thalassochelydians, including *Solnhofia parsonsi* (NMS 8741 and JM SCHA 70). Therefore, they were likely present in *Solnhofia brachyrhyncha* as well, but the relatively poor preservation of the region of the apertura narium externa in MJSN BAN001-2.1 prevents any confirmation. Fragments of bones lying anterior to the prefrontals might correspond to remnants of the nasals (Figs. 3A and 3B). The nasal region is missing in MJSN SCR010-1214 (Fig. 5).

#### Prefrontal

The prefrontals are badly preserved in MJSN BAN001-2.1 and missing in MJSN SCR010-1214. The right prefrontal is broken into several pieces, but appears to be a roughly quadrangular element contacting the nasal anteriorly, the ascending process of the maxilla anterolaterally, the frontal posteriorly, and the other prefrontal medially along its entire length (Fig. 3A and 3B). The left prefrontal is preserved as an elongate element forming the anterodorsal margin of the orbit. The anterior and medial parts of the left prefrontal are damaged. Only the posterior contact with the left frontal and part of the medial contact with the right prefrontal are preserved. The posterior suture with the frontal and the anterior suture with the nasal were probably mostly transverse, but it is difficult to be sure. The descending process of the prefrontal is broken on both sides, so nothing can be said about the contacts of the prefrontal in the anterior part of the fossa orbitalis and region of the foramen orbito-nasale.

#### Frontal

The frontals are large elements forming most of the dorsal margin of the orbits. Each of the frontals contacts the prefrontal anteriorly, the postorbital posterolaterally, the parietal posteriorly, and its counterpart medially (Fig. 3A and 3B). The suture with the parietal is transverse and strongly interdigitating. The suture with the postorbital is V-shaped and concave relative to the frontal, which reminds the condition in *Solnhofia parsonsi* (TM 4023 and NMS 8741). The anterior suture with the prefrontal is probably transverse, but the preservation in that area is imperfect. In many thalassochelydians, including *Solnhofia parsonsi*, the frontals extend anteromedially below the prefrontal and contact the nasals in the roof of the fossa nasalis. The state of preservation of MJSN BAN001-2.1 prevents us from telling whether this is also a condition present in *Solnhofia brachyrhyncha*.

#### Parietal

On the skull roof, the parietal meets the frontal anteriorly, the postorbital laterally and the other parietal medially (Figs. 3A and 3B). In both crania, the posterior part of the bone is shattered in many fragments and it is unclear whether the parietal contacted the squamosal behind the postorbital, although it seems unlikely (see Squamosal). Of the posterior margin of the parietal, only the anteriormost embayment of the right upper temporal emargination is preserved in MJSN BAN001-2.1 (Figs. 3A and 3B). The development and shape of the upper temporal emargination appear to be similar to what is known (also imperfectly) in *Solnhofia parsonsi* (NMS 8741 and MNHN CNJ 82).

As usual, the parietals form the anterior half of the roof of the cavum cranii and send a well-developed process ventrally to form the lateral braincase wall. Like in *Solnhofia parsonsi*, the parietal contributes significantly to the large processus trochlearis oticum (Figs. 3A–3D, 4A, 4B and 5A–5D). Evers & Joyce (2020) recently described a similar condition in the sandownid *Sandownia harrisi*, but the contribution of the parietal to the processus trochlearis oticum in that taxon is much more reduced than in *So. parsonsi* and *So. brachyrhyncha*. The descending process of the parietal forms most of the posterior and anterior margins of the foramen nervi trigemini (Figs. 4A and 4B). The ventral margin of the foramen nervi trigemini is formed by the pterygoid (see Pterygoid). In *So. parsonsi* (TM 4023), the contribution of the parietal is limited to the dorsal margin of the foramen nervi trigemini, whereas the pterygoid forms the anteroventral, ventral and most of the posterior margins of the foramen (Fig. 4C and 4D). Posterior to the foramen nervi trigemini, the parietal has a broad contact with the pterygoid in *So. brachyrhyncha*, clearly excluding the prootic from the posterior margin of the foramen nervi trigemini (Figs. 4A and 4B). In *So. parsonsi* (TM 4023), the parietal-pterygoid contact is much more reduced and the prootic extends closer to the posterior margin of the foramen nervi trigemini (Figs. 4C and 4D). Anterior to the foramen nervi trigemini, the descending process of the parietal and the pterygoid are fully separated by the epipterygoid in lateral view, but there is apparently a parietal-pterygoid point contact medial to the epipterygoid along the anterior margin of the foramen nervi trigemini (Figs. 4A and 4B). It is unknown whether this contact is more extensive on the medial surface of the lateral braincase wall. In *So. parsonsi* (TM 4023), the epipterygoid apparently fully separates the parietal and pterygoid in both lateral and medial views.

#### Jugal

The jugal is only preserved in MJSN BAN001-2.1, but it is damaged on both sides so that the limits and contacts of the bone are somewhat unclear. It appears that the jugal forms the posteroventral margin of the orbit and contacts the postorbital dorsally, the maxilla anteroventrally, and the pterygoid posteroventrally (Figs. 3A–3D). As in *Solnhofia parsonsi* (TM 4023, NMS 8741, JM SCHA 70), the jugal also has an anteromedial contact with the palatine in the floor of the fossa orbitalis, but in contrast to the aforementioned species the jugal does not extend as far anteriorly as the level of the foramen orbito-nasale in *Solnhofia brachyrhyncha* (Figs. 3A and 3B). As in *So. parsonsi* (TM 4023) and *Sandownia harrisi* (Evers & Joyce, 2020), the medial process of the jugal forms a long posteromedial extension along the posteromedial border of the maxilla and the anterolateral border of the pterygoid (Figs. 3C and 3D). As preserved in MJSN BAN001-2.1, this posteromedial extension of the jugal is partly visible in ventral view, and appears to contact the maxilla and pterygoid laterally. However, parts of the bone still in place indicate that this extension of the jugal was in contact with the dorsal surface of the maxilla and pterygoid and therefore was not visible in ventral view, similar to *So. parsonsi* (TM 4023). This differs from *Sa. harrisi* where the posteromedial extension of the medial process of the jugal extends laterally to the pterygoid and contributes to the secondary palate (Evers & Joyce, 2020). The more lateral part of the medial process of the jugal forms a vertical ridge that partly delimits the fossa orbitalis posteriorly and extends dorsally on the medial surface of the main part of the jugal, as preserved on the right side of MJSN BAN001-2.1 (see 3D Model; Additional Information; Anquetin & Püntener (2020)). A similar ridge is present in *So. parsonsi* (TM 4023, NMS 8741) and *Sa. harrisi* (Evers & Joyce, 2020). Posteriorly, the medial process of the jugal reaches the anterodorsal corner of the processus pterygoideus externus. The medial process of the jugal reaches as far posteriorly in *So. parsonsi* (TM 4023, NMS 8741), but does not really contact the more reduced processus pterygoideus externus (see Pterygoid).

#### Quadratojugal

In MJSN BAN001-2.1, only a very small fragment of the left quadratojugal is preserved along the anterior margin of the cavum tympani (Figs. 3A–3D and 3G). In MJSN SCR010-1214, more of the quadratojugal is preserved, but the bone is quite fragmented (Figs. 5A–5D and 5F). As preserved, the quadratojugal contacts the squamosal above the cavum tympani and the quadrate ventrally and medially. The quadratojugal appears to brace the cavum tympani anteriorly without entering its margin. Dorsally, there is apparently a contact between the quadratojugal and the postorbital, but this area is much fragmented on both sides. As in *Solnhofia parsonsi* (TM 4023) and most sandownids (Evers & Joyce, 2020), the quadratojugal forms the lateralmost part of the extensively developed processus trochlearis oticum.

#### Squamosal

Except maybe for a small fragment on the right side (Figs. 3A and 3B), the squamosal is entirely missing in MJSN BAN001-2.1. The two squamosals are present in MJSN SCR010-1214, but they are incompletely preserved and broken into several pieces (Fig. 5). The squamosal contacts the quadratojugal anterolaterally above the cavum tympani, the quadrate anteriorly and ventrally, and the opisthotic anteromedially. As in most turtles, the squamosal apparently formed the antrum postoticum, although this area is poorly preserved here. The posterior process of the squamosal forms a subvertical (or slightly oblique) lamina extending far behind the level of the occipital plane (Fig. 5). This apparently corresponds to what is known in *Solnhofia parsonsi* (TM 4023, NMS 8741, JM SCHA 70), although the posteriormost part of the squamosal is not fully preserved or accessible in any of these specimens. Anterodorsally, the lamina curves medially and forms the posterolateral margin of the upper temporal emargination. More dorsally and anteriorly, the squamosal is broken on either side so that the contacts with other bones in that area remain unclear. However, it seems unlikely that the squamosal contacted the parietal in *Solnhofia brachyrhyncha*.

#### Postorbital

The postorbital is incompletely preserved in both of the available crania. In MJSN BAN001-2.1, only the anteriormost part of the left postorbital is preserved, while the right postorbital is represented by a larger portion extending from the orbit to the anteriormost part of the upper temporal emargination (Figs. 3A, 3B and 3F). A comparable portion of the left postorbital is preserved in MJSN SCR010-1214, but its dorsal aspect is heavily fragmented (Figs. 5A–5D). A triangular, fragmented piece of bone lying on top of the right processus trochlearis oticum probably corresponds to the posterior part of the right postorbital, but it is impossible to confirm. The postorbital forms the posterodorsal margin of the orbit. As preserved, the postorbital contacts the frontal anteromedially, the parietal medially, and the jugal ventrally. A contact with the quadratojugal was probably also present posteroventrally (see left side of MJSN SCR010-1214; Figs. 5A and 5B), but the area is poorly preserved. A strong ridge runs on the medial surface of the postorbital (Figs. 5C and 5D). A similar ridge is present in *Solnhofia parsonsi* (TM 4023, NMS 8741) and *Sandownia harrisi*, and continues on the medial surface of the jugal (Evers & Joyce, 2020). Therefore, it appears likely that the ridges observed on the medial surface of the postorbital (MJSN SCR010-1214) and jugal (MJSN BAN001-2.1) of *Solnhofia brachyrhyncha* were also continuous.

#### Premaxilla

The premaxilla is poorly known in *Solnhofia brachyrhyncha*. Only the palatal part of the two premaxillae is preserved in MJSN BAN001-2.1 (Figs. 3C and 3D). There, the premaxilla contacts the maxilla laterally and the other premaxilla medially. The vomer is missing in that specimen, so nothing can be said about the relation beween this bone and the premaxilla, or the presence/absence of the foramen preapalatinum. More anterior portions of the premaxillae such as their contribution to the labial ridge and to the ventral border of the apertura narium externa are entirely missing. It is therefore impossible to tell whether the premaxillae projected anteriorly as they do so characteristically in *Solnhofia parsonsi* (TM 4023). In the latter, the palatal part of the premaxilla is flat and contributes fully to the triturating surface. In *Solnhofia brachyrhyncha*, the palatal part of the premaxilla is apparently divided into a flat lateral surface continuous with the triturating surface of the maxilla and a dorsally arched medial surface delimiting a midline trough. However, this observation may as well be the result of deformation and disarticulation in MJSN BAN001-2.1. More material is needed to confirm or contradict this potential difference between *So. brachyrhyncha* and *So. parsonsi*.

#### Maxilla

The maxilla is only preserved in MJSN BAN001-2.1. It contacts the premaxilla anteromedially, the prefrontal (and probably also the nasal) anterodorsally, the jugal posterodorsally, the pterygoid posteriorly, and the palatine posteromedially (Figs. 3A–3D). The maxilla probably also contacted the vomer medially, but this bone is lost in MJSN BAN001-2.1 (see Vomer). The maxilla forms the ventral and anteroventral margins of the orbit. As noted above, the jugal does not extend as far anteriorly in the floor of the fossa orbitalis as it does in *Solnhofia parsonsi*. As a result, the maxilla forms a larger portion of the floor of the fossa orbitalis in *So. brachyrhyncha*. Since the ventral process of the prefrontal is damaged on both sides, the foramen orbito-nasale is not readily observable in MJSN BAN001-2.1. However, the foramen alveolare superius, which opens just medial to the lateral margin of the foramen orbito-nasale in most turtles including *So. parsonsi*, is visible in the left side of that specimen. In many thalassochelydians including *Plesiochelys etalloni*, *Portlandemys mcdowelli* and *Jurassichelon oleronensis* (Gaffney, 1976; Anquetin, Püntener & Billon-Bruyat, 2015), as well as *So. parsonsi* (TM 4023, NMS 8741), a small unnamed foramen is present lateral to the foramen orbito-nasale just inside (as it is the case for *So. parsonsi*) or outside of the anteroventral corner of the orbit. This area is damaged in each side of MJSN BAN001-2.1, so that the presence or absence of this unnamed foramen in *So. brachyrhyncha* cannot be elucidated. However, a foramen occurs more posteriorly in the floor of the fossa orbitalis approximately at midway along the ventral margin of the orbit. This foramen is only visible in the right side of MJSN BAN001-2.1, but its presence on the left side cannot be confirmed as the corresponding area is damaged and partly covered by matrix. This foramen may correspond to the unnamed foramen of other thalassochelydians.

As in *So. parsonsi* (TM 4023, NMS 8741, JM SCHA 70), the secondary palate of *So. brachyrhyncha* is formed primarily by the maxillae (Figs. 3C and 3D). The following characteristics are also similar in the two species: the labial ridge is acute, moderately high, and lined with moderately sized nutritive foramina; the triturating surface itself is broadly arched dorsally and pierced by a few large nutritive foramina; the lingual ridge is nonexistent. However, the shape of the triturating surface is different. In *So. parsonsi* (TM 4023, NMS 8741, JM SCHA 70), the triturating surface is V-shaped with straight lateral margins, and the width of each ramus of the V remains the same anteroposteriorly. The triturating surface is also much elongated in this species in relation with the great elongation of the snout. In *So. brachyrhyncha*, each ramus of the triturating surface is very wide posteriorly and reduces much in width anteriorly, notably from the level of the middle of the orbit. As a result, the lateral margins of the triturating surface are slightly sinusoidal in ventral view, the anterior half being concave laterally and the posterior half concave medially (Figs. 3C and 3D).

#### Vomer

The vomer is apparently disarticulated and lost in MJSN BAN001-2.1 (Figs. 3C and 3D). A minute fragment of bone between the two maxillae may be a remnant of the vomer, but reveals nothing noteworthy. The palate, including the vomer, is entirely missing in MSJN SCR010-1214.

#### Palatine

The palatines are preserved only in MJSN BAN001-2.1, but their state of preservation is quite poor (Figs. 3A–3D). The palatine contacts the maxilla anterolaterally, the jugal laterally in the floor of the fossa orbitalis, and the pterygoid posteriorly. Anterior contacts with the vomer and descending process of the prefrontal were probably also present, but these elements are lost or damaged in that specimen. The palatine borders the apertura narium interna and forms the roof of the narial passage. In *Solnhofia parsonsi* (TM 4023, NMS 8741, JM SCHA 70), the palatine contributes to the triturating surface and forms an incomplete floor to the narial passage. This contribution of the palatine to the triturating surface and partial flooring of the narial passage are absent in *So. brachyrhyncha* (Figs. 3C and 3D). The posterior half of the palatine is poorly preserved, but zigzagging sutural marks on the pterygoid indicate that the palatine extended as far posteriorly as the posterior part of the processus pterygoideus externus, which reminds the condition in *So. parsonsi* (TM 4023, JM SCHA 70). The foramen palatinum posterius (only preserved on the left side of MJSN BAN001-2.1) is a small oval opening located anterior to the level of the processus pterygoideus externus. As preserved, the foramen palatinum posterius appears to be formed by the palatine medially and the pterygoid laterally. In *So. parsonsi* (TM 4023), the foramen palatinum posterius consists in a short canal formed primarily by the palatine with a modest lateral contribution from the pterygoid, but the ventral opening of this canal is formed entirely by the palatine (Evers & Joyce, 2020). However, this is not readily observable from external examination because previous authors have described the foramen palatinum posterius as being formed by the palatine and pterygoid in *So. parsonsi* (Parsons & Williams, 1961, Gaffney, 1975; Joyce, 2000). Therefore, we consider that the state of preservation of MJSN BAN001-2.1 prevents proper comparison between *So. brachyrhyncha* and *So. parsonsi* regarding which bones form the foramen palatinum posterius.

#### Quadrate

The quadrate can be observed in both MJSN BAN001-2.1 and MSJN SCR010-1214, but with variable degrees of preservation depending on specimen and side (Figs. 3–5). As usual, the quadrate forms most of the lateral part of the processus trochlearis oticum (with a lateral contribution from the quadratojugal), the posterolateral half of the foramen stapedio-temporale, the cavum tympani and the incisura columellae auris (which remained open posteroventrally), and the condylus mandibularis. In dorsal view, the quadrate contacts the prootic anteromedially, the opisthotic posteromedially, the squamosal posteriorly, and the quadratojugal laterally (Figs. 3A, 3B, 5A and 5B). Below the processus trochlearis oticum, the quadrate contacts the prootic anteromedially, the pterygoid medially and ventrally, and the quadratojugal laterally (Figs. 3C, 3D, 4, 5C and 5D). In the roof of the cavum acustico-jugulare and posterior to the incisura columellae auris, the quadrate contacts the prootic anteromedially (forming the aditus canalis stapedio-temporalis), the opisthotic posteromedially, and the squamosal posterolaterally (Figs. 3C, 3D, 5C and 5D). The quadrate contributes more to the processus trochlearis oticum than in *Solnhofia parsonsi* (TM 4023, NMS 8741) to the detriment of the prootic (Fig. 4; see Prootic). The condylus mandibularis is best preserved in the left side of MJSN BAN001-2.1, but the condyle is not complete. The articular surface appears to be just slightly concave. As in *So. parsoni* (TM 4023, JM SCHA 70), the lateral lobe of the condyle articular surface seems to be larger than the medial lobe. The condylus mandibularis is located anterior to the level of the occipital plane in *So. brachyrhyncha* as in most other turtles, which differs from the condition in *So. parsonsi* (TM 4023, NMS 8741, JM SCHA 70) where the condylus mandibularis is situated at the level of the occipital plane. As in all thalassochelydians, and to a lesser extent sandownids, a strong ventrally infolding ridge is developed on the posterior surface of the quadrate articular process below the incisura columellae auris. This ridge prolongates the posterior margin of the pterygoid laterally, passes below the incisura columellae auris, then bends strongly ventrally and terminates at the lateroventral corner of the mandibular process of the quadrate (see 3D Model; Additional Information; Anquetin & Püntener (2020)).

#### Epipterygoid

The epipterygoid is only preserved in MJSN BAN001-2.1. It contacts the parietal dorsally and the pterygoid ventrally (Figs. 4A and 4B). It is possible that the ventral part of the bone is broken revealing the crista pterygoidea medial to it. But in any case, the lateral exposure of the epipterygoid is more limited in *Solnhofia brachyrhyncha* reaching only as high as the center of the foramen nervi trigemini, whereas it extends as high as the top of the foramen nervi trigemini in *So. parsonsi* (TM 4023). The contribution of the epiterygoid to the anterior margin of the foramen nervi trigemini appears to be limited to a point contact (Figs. 4A and 4B), but this may be an artefact due to the breaking of the lower part of the bone. In *So. parsonsi* (TM 4023), the contribution of the epipterygoid to the anterior margin of the foramen nervi trigemini is more extensive and located more dorsally along the anterior margin of the foramen (Figs. 4C and 4D). As described above (see Parietal), the parietal and pterygoid are contacting one another medial to the epipterygoid along the anterior margin of the foramen nervi trigemini, but it is unknown whether this contact is more extensive on the medial aspect of the lateral braincase wall. In *So. parsonsi* (TM 4023), the epipterygoid fully separates the parietal from the pterygoid in both lateral and medial view. Finally, *So. brachyrhyncha* lacks the well-developed blunt ventral process on the lateral surface of the epipterygoid that is present in *So. parsonsi* (TM 4023, NMS 8741; Figs. 4C and 4D).

#### Pterygoid

The pterygoid is best preserved in MJSN BAN001-2.1 (Figs. 3A–3D, 4A and 4B). In MJSN SCR010-1214, only a part of the ventral aspect of the pterygoid is present (Figs. 5C and 5D). In ventral view, the pterygoid contacts the jugal anterodorsally, the maxilla anterolaterally, the palatine anteriorly, the other pterygoid medially for its anterior half, the parabasisphenoid medially for its posterior half, and the quadrate posterolaterally. The suture between the parabasisphenoid and basioccipital is not clear in MJSN BAN001-2.1 so it is uncertain whether the pterygoid contacted the basioccipital in *Solnhofia brachyrhyncha*. The condition in *So. parsonsi* is actually somewhat ambiguous. Based on external examination previous authors have described a pterygoid-basioccipital contact in this species (Parsons & Williams, 1961, Gaffney, 1975; Joyce, 2000). However, CT scan data reveal that the parabasisphenoid is intercalated between the pterygoid ventrally and the basioccipital dorsally in TM 4023 (Evers & Benson, 2018). If present, the contact between the pterygoid and basioccipital is probably limited. As much as can be interpreted from MJSN BAN001-2.1, the condition in *So. brachyrhyncha* does not appear to be much different from the one in *So. parsonsi* (Figs. 3C and 3D).

Anteriorly, the suture with the palatine is somewhat unclear, but zigzagging marks on the pterygoid indicate the posterior extension of the sutural contact with the palatine (see Palatine). In *So. parsonsi* (TM 4023, NMS 8741, JM SCHA 70), the choanae extend much posteriorly over the pterygoids well beyond the level of the processus pterygoideus externus. In *So. brachyrhyncha*, the choanae are limited to the palatine as common in most turtles (Figs. 3C and 3D). In *So. parsonsi* (TM 4023, NMS 8741, JM SCHA 70), the processus pterygoideus externus is reduced to a small nubbin. In *So. brachyrhyncha*, the processus pterygoideus externus is more developed, albeit much reduced compared to most turtles. The processus pterygoideus externus is a short process ovoid in section projecting laterally from the pterygoid (Figs. 3C, 3D, 4A and 4B). As noted above (see Jugal), the anterodorsal corner of the processus pterygoideus externus contacts the posterior tip of the medial process of the jugal. Although the jugal reaches as posteriorly in *So. parsonsi* (TM 4023, NMS 8741), it does not contact the much reduced processus pterygoideus externus. An arched ridge runs on the ventral surface of the pterygoid from the posterior part of the processus pterygoideus externus to the posterior limit of the bone very close to the suture with the parabasisphenoid. In constrast to *So. parsonsi* (TM 4023, NMS 8741, JM SCHA 70), this ridge is weakly developed in *So. brachyrhyncha*.

The foramen posterius canalis carotici interni opens in the posteromedial corner of the pterygoid and is apparent in ventral view, as preserved (Figs. 3C and 3D). However, this area is somewhat damaged in the two available specimens. In *So. parsonsi* (TM 4023), the foramen posterius canalis carotici interni opens slightly more posteriorly and dorsal to the arched ridge running on the ventral surface of the pterygoid, so that the foramen is partly hidden in ventral view. Therefore, the condition in the two species is maybe slightly different. On the left side of MJSN BAN001-2.1, a small foramen opens a short distance anterior to the foramen posterius canalis carotici interni, just medial to the arched ridge running on the pterygoid and approximately halfway along the suture between the pterygoid and parabasisphenoid (Figs. 3C and 3D). A similar foramen is present in *So. parsonsi* (TM 4023, NMS 8741). The nature of this foramen is controversial (Gaffney, 1975). The pterygoid fossa is similarly deep in *So. parsonsi* (TM 4023, NMS 8741) and *So. brachyrhyncha*, but the fossa is more pinched mediolaterally in the former species.

Below the processus trochlearis oticum and in the ethmoid region, the pterygoid contacts the quadrate posterolaterally, the prootic posterodorsally, the parietal posteromedially, and the epipterygoid anteromedially (Figs. 4A and 4B). Compared to *So. parsonsi* (TM 4023; Figs. 4C and 4D), the contact of the pterygoid with the prootic and its contribution to the foramen nervi trigemini are reduced due to the greater posteroventral extension of the parietal behind the foramen nervi trigemini. The pterygoid forms the ventral margin of the foramen nervi trigemini. As noted above (see Parietal), the pterygoid apparently also contacts the parietal anterior to the foramen nervi trigemini and medial to the epipterygoid. The extent of this contact on the medial surface of the lateral braincase wall is uncertain.

#### Supraoccipital

The supraoccipital can be observed in both MJSN BAN001-2.1 and MJSN SCR010-1214 (Figs. 3 and 5). It contacts the parietal anterodorsally, the prootic anterolaterally, the opisthotic posterolaterally, and the exoccipital posteroventrally. A contact between the prootic and opisthotic prevents the supraoccipital from contacting the quadrate posterior to the foramen stapedio-temporale. The contacts of the supraoccipital are similar in *Solnhofia parsonsi* (TM 4023). The crista supraoccipitalis extends greatly posterior to the occipital plane. The posterior tip of the crista supraoccipitalis is apparently missing in MJSN SCR010-1214, but the crista reaches almost as far posteriorly as the squamosals (Figs. 5A–5D). This also corresponds to the condition in *So. parsonsi* (JM SCHA 70).

#### Exoccipital

The exoccipitals are best preserved in MJSN BAN001-2.1 (Figs. 3A–3D). The exoccipital contacts the supraoccipital dorsally, the opisthotic laterally and the basioccipital ventrally. There is no contact between the exoccipital and pterygoid. The exoccipital forms the lateral margin of the foramen magnum and the dorsolateral lobe of the three-lobed condylus occipitalis. The exoccipitals appear to meet one another in the ventral margin of the foramen magnum. Ventrally, the exoccipital is pierced by a pair of foramina nervi hypoglossi. This morphology is similar to that of *Solnhofia parsonsi* (TM 4023).

#### Basioccipital

The basioccipital is only observable in MJSN BAN001-2.1, but its preservation is rather poor (Figs. 3A–3D). It contacts the parabasisphenoid anteriorly along a more or less transverse suture and the exoccipital dorsally. An anterolateral contact with the pterygoid was possibly also present on the ventral aspect of the skull, but cannot really be confirmed based on the available material (see Pterygoid). The ventral exposure of the basioccipital is somewhat quadrangular and wider than long. However, the ventral surface of the bone is eroded in MJSN BAN001-2.1. The basioccipital forms the ventral lobe of the three-lobed condylus occipitalis.

#### Prootic

The prootic can observed in both MJSN BAN001-2.1 (Figs. 3, 4A and 4B) and MJSN SCR010-1214 (Fig. 5). In the dorsal surface of the otic chamber, the prootic contacts the parietal anteromedially, the supraoccipital posteromedially, the opisthotic posteriorly, and the quadrate posterolaterally. It forms the anteromedial half of the foramen stapedio-temporale. Medial to this foramen, the dorsal surface of the prootic is deeply incised by a medially oriented trough marking the passage of the arteria stapedialis.

Below the processus trochlearis oticum, the prootic contacts the quadrate laterally, the pterygoid ventrally, and the parietal medially. As noted for most thalassochelydians (Evers & Benson, 2019), the processus trochlearis oticum is bordered laterally by a deep recess (Figs. 3 and 5). As in *Solnhofia parsonsi* (TM 4023, NMS 8741, JM SCHA 70) and sandownids, the processus trochlearis oticum of *Solnhofia brachyrhyncha* is greatly developed and projecting much forward, hiding the foramen nervi trigemini in lateral view. However, a number of differences can be observed between the two species of *Solnhofia*. In dorsal or ventral view, the processus trochlearis oticum is more oblique in *So. brachyrhyncha*, whereas it is oriented more transversely in *So. parsonsi*. The contribution of the prootic to the processus trochlearis oticum is reduced in *So. brachyrhyncha* compared to *So. parsonsi* (Fig. 4). As noted above, the quadrate and the parietal (especially ventrally) contribute more to the processus trochlearis oticum in *So. brachyrhyncha*. Finally, the processus trochlearis oticum, notably the part formed by the prootic, is not as deeply concave in *So. brachyrhyncha* (Fig. 4). In *So. parsonsi* (TM 4023), the prootic is prevented from entering the posterior margin of the foramen nervi trigemini only by a short contact between the parietal and pterygoid (Figs. 4C and 4D). In *So. brachyrhyncha*, the parietal-pterygoid contact is much broader and the prootic is more clearly excluded from the posterior margin of the foramen nervi trigemini in lateral view (Figs. 4A and 4B).

In the cavum acustico-jugulare, the prootic contacts the pterygoid ventrally, the opisthotic dorsomedially, and the quadrate dorsolaterally. There was probably also a medioventral contact with the parabasisphenoid, but this contact is not preserved in MJSN BAN001-2.1. The prootic forms the medial half of the aditus canalis stapedio-temporalis and the roof of the posterior part of the canalis cavernosus, the latter opening just ventromedial to the former. The morphology of this area is similar in *So. parsonsi* (TM 4023).

#### Opisthotic

The opisthotic contacts the exoccipital posteromedially, the supraoccipital anteromedially, the prootic anteriorly, the quadrate laterally, and the squamosal posterolaterally (Figs. 3 and 5). The anterior contact with the prootic prevents the quadrate from meeting the supraoccipital on the dorsal surface of the otic chamber. The paroccipital process is oriented mostly posteriorly and extends much beyond the level of the occipital plane, more so in ventral aspect. The occipital part of the opisthotic forms a broad, moderately concave triangular surface delimited dorsally and ventrally by a pronounced, vertical ridge. This is similar to the condition in *Solnhofia parsonsi* (TM 4023), but differs for example from the rod-like structure observed in *Plesiochelys planiceps* or the ridge-like structure in *Sandownia harrisi* (Evers & Joyce, 2020). In the cavum acustico-jugulare, the opisthotic contacts the exoccipital posteromedially, the prootic anteriorly, and the quadrate laterally. The processus interfenestralis is only preserved (partly) on the right side of the MJSN BAN001-2.1, the side most affected by deformation. It seems that the processus interfenestralis does not contact the pterygoid or the basioccipital ventrally, which is also the condition in *So. parsonsi* (TM 4023). The foramen externum nervi glossopharyngei pierces the dorsal base of the processus interfenestralis of the opisthotic close to its lateral margin.

#### Parabasisphenoid

The parabasisphenoid can be observed only in MJSN BAN001-2.1 (Figs. 3C and 3D). Only the ventral aspect of the bone can be seen because the interior of the cranium is filled up with matrix. The parabasisphenoid contacts the pterygoid anteriorly and laterally, and the basioccipital posteriorly. The ventral exposure of the parabasisphenoid is triangular and more elongated than in *Sonhofia parsonsi* (TM 4023, NMS 8741, JM SCHA 70). Anteriorly, the parabasisphenoid reaches almost the level of the posterior part of the processus pterygoideus externus in *So. brachyrhyncha*, whereas it barely goes beyond the level of the anterior limit of the pterygoid fossa in *So. parsonsi* (TM 4023, NMS 8741).

### Mandible

#### General description

**Figure 6:**
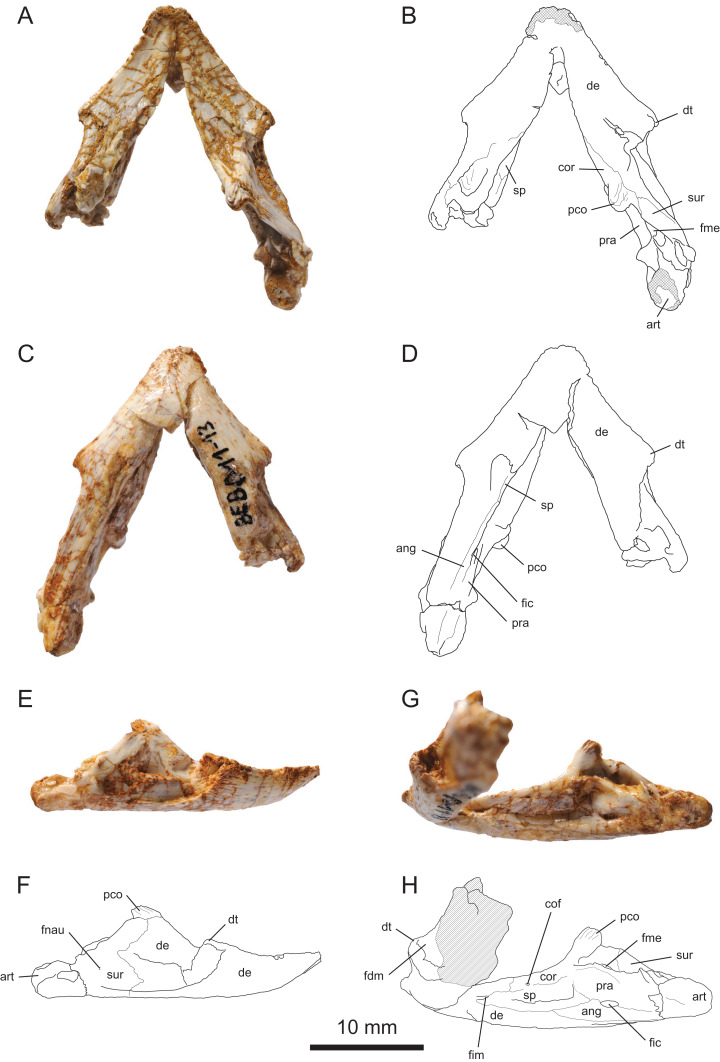
MJSN BEB011-13, mandible of *Solnhofia brachyrhyncha* (Kimmeridgian, Porrentruy, Switzerland). (A) Mandible in dorsal view; (B) interpretative drawing of the mandible in dorsal view; (C) mandible in ventral view; (D) interpretative drawing of the mandible in ventral view; (E) mandible in right lateral view; (F) interpretative drawing of the mandible in right lateral view; (G) right ramus of the mandible in lingual view; (H) interpretative drawing of the right ramus of the mandible in lingual view. In the drawings, hatchings correspond to damaged areas, whereas the criss-cross pattern indicates areas heavily covered by iron oxides. Abbreviations: ang, angular; art, articular; cof, coronoid foramen; cor, coronoid; de, dentary; dt, dentary tubercle; fdm, foramen dentofaciale majus; fic, foramen intermandibularis caudalis; fim, foramen intermandibularis medius; fme, fossa meckelii; fnau, foramen nervi auriculotemporalis; pco, processus coronoideus; pra, prearticular; sp, splenial; sur, surangular.

MJSN BEB011-13 is a relatively small mandible (Fig. 6). The right ramus is complete, but the left one is broken at the level of the anterior margin of the fossa meckelii and the posterior portion of this ramus is missing. The mandible suffered some deformation during fossilization: the dorsal aspect of the mandible is shifted slightly to the left compared to the ventral aspect. The bones are crisscrossed by many small fractures that sometimes complicate the interpretation of sutures. These fractures and other areas, such as the anterior tip of the symphysis and the right articular surface are encrusted by iron oxides (Fig. 6).

The anterior part of the mandible has a relatively flat and low profile (Figs. 6E and 6F). Posteriorly, the processus coronoideus is greatly developed. This somewhat reminds the condition in *Solnhofia parsonsi* (TM 4023). However, the outline of the mandible in dorsal or ventral view is remarkable by the presence of large dentary tubercles projecting laterally on each side. This is radically different from the mandible of *So. parsonsi* (TM 4023, JM SCHA 70), which is V-shaped and has straight sides (Parsons & Williams, 1961; Gaffney, 1975; Joyce, 2000). With a total length of 27.75 mm and a maximal width of 26.3 mm (Table 2), the mandible MJSN BEB011-13 is about as wide as long, which contrasts with the clearly longer-than-wide mandible of *So. parsonsi* (TM 4023, JM SCHA 70).

**Table 2:** Measurements of crania and mandibles referred to *Solnhofia* spp. Measurements for TM 4023, JM SCHA 70 and ICHLU 005.4.20 were taken from Gaffney (1975), Joyce (2000), and Lapparent de Broin, Lange-Badré & Dutrieux (1996), respectively. Measurements in parentheses are estimates that were derived indirectly from a 3D model for TM 4023 and photographs for JM SCHA 70. Maximum skull length was measured either at the level of the crista supraoccipitalis or at the level of the maximal posterior extension of the squamosals, depending on the preservation of each specimen.

	*Solnhofia parsonsi*			*Solnhofia* sp.	*Solnhofia* aff. *parsonsi*	*Solnhofia brachyrhyncha*	
	TM 4023	NMS 8741	JM SCHA 70	MNHN CNJ 82	ICHLU 005.4.20	MJSN BAN001-2.1	MJSN BEB011-13
Skull length (mm)	73	58.2	59	49.3		53.3	
Skull length, max (mm)		70.3	71	55.9		64.2	
Skull width, max (mm)	(52.8)	45.3	40	35	60	48.75	
Mandible length (mm)	(62.7)		(59.2)				27.75
Mandible width (mm)	(47.5)		(41.5)				26.3
Symphysis length (mm)	(27.8)		(24.4)				7.65

#### Dentary

The dentary contacts the coronoid posterodorsally, the splenial posteromedially, the angular posteroventrally, and the surangular posterolaterally (Fig. 6). The dentaries are fused anteriorly and form a short symphysis. The symphysis represents about 41–44% of the length of the mandible in *Solnhofia parsonsi* (TM 4023, JM SCHA 70), whereas it represents only 27.6% of the mandible length in *Solnhofia brachyrhyncha* (Table 2). In *So. parsonsi* (TM 4023, JM SCHA 70), the triturating surface extends for the total length of the symphysis. In contrast, the triturating surface only covers the anterior half of the symphysis in *So. brachyrhyncha* (Figs. 6A and 6B). The posterior half of the symphysis consists of a posteroventrally slopping furrow between the two mandibular rami. The dentaries form most of the triturating surface, with a small posteromedial contribution from the coronoids. As in *So. parsonsi* (TM 4023), the triturating surface is flat and bears no labial or lingual ridge. In lateral view, the anterior tip of the mandible is slightly turned upward. Each ramus of the triturating surface widens posteriorly and reaches its greatest width at the level of a large, laterally projecting dentary tubercle (Figs. 6A and 6B). In *So. parsonsi* (TM 4023, JM SCHA 70), the triturating surface is wider anteriorly because of the extended symphysis, and there is no trace of a dentary tubercle.

The greatly developed dentary turbercles of *So. brachyrhyncha* are highly distinctive among thalassochelydians. Comparable structures occur in other more or less distantly related taxa, notably in some sandownids like *Sandownia harrisi* (Meylan et al., 2000; Evers & Joyce, 2020) and possibly *Brachyopsemys tingitana* (Tong & Meylan, 2013). Dentary tubercles are also developed in some baenids (Archibald & Hutchison, 1979; Gaffney, 1982; Lyson & Joyce, 2009a, 2009b), some bothremydid pleurodires (Gaffney, Tong & Meylan, 2006), as well as kinosternids (Bourque, 2013). The dentary tubercle probably served for muscular attachment. In *So. brachyrhyncha*, the dentary tubercle could also be a way to expand the width of the posterior part of the triturating surface on the mandible, matching the shape of the upper triturating surface (Figs. 3 and 6).

Posterior to the large dentary tubercle, the lateral surface of the dentary is deeply recessed. The foramen dentofaciale majus apparently opens within that recess, but it is difficult to locate it with precision in MJSN BEB-011-13 (Figs. 6E–6H). Posterolaterally, the dentary has a long zigzagging suture with the surangular, but the length and shape of the posteroventral extension of the dentary between the surangular and the angular are unknown because the sutures are poorly preserved in that area (Figs. 6C and 6D). On the medial surface of the mandibular ramus, the dentary forms the anterolateral margin of the relatively small foramen intermandibularis medius, the other half being formed only by the splenial. This foramen is located much anteriorly due to the long anterior extension of the splenial. The foramen intermandibularis medius actually opens just behind the level of the most posterior part of the mandibular symphysis. In *So. parsonsi* (TM 4023), the foramen intermandibularis medius is located more posteriorly (but still just behind the level of the the posterior part of the symphysis) and formed by the dentary, the splenial and the coronoid.

#### Angular

The right angular is apparently mostly complete in MJSN BEB011-13, but only the sutures on the medial surface of the right ramus can be traced with some certainty (Figs. 6G and 6H). On the medial surface of the ramus, the angular contacts the dentary anteroventrally, the splenial anterodorsally, the prearticular dorsally, and the articular posteriorly. An oval foramen intermandibularis caudalis opens in the anterior part of the suture between the angular and prearticular level with the anterior part of the fossa meckelii. In *Solnhofia parsonsi* (TM 4023), the proportionally smaller foramen intermandibularis caudalis is located more posteriorly (level with the posterior part of the fossa meckelii) and may have been formed mostly by the angular.

#### Surangular

The surangular forms the posterior third of the lateral surface of the mandible (Figs. 6E and 6F) as well as the lateral wall of the fossa meckelii (Figs. 6A and 6B). The surangular contacts the dentary anteriorly, the coronoid anterodorsally, and the articular posterodorsally. There was probably also a ventromedial contact with the angular, but sutures are difficult to see in that area. A small foramen nervi auricolotemporalis opens in the middle of the bone (Figs. 6E and 6F). The mandibular ramus is very high at the level of the processus coronoideus. As a result, the anterodorsal part of the surangular projects much dorsally and reaches the base of the processus coronoideus. In *Solnhofia parsonsi* (TM 4023), the surangular does not reach as high anterodorsally and the coronoid is exposed posteroventral to the processus coronoideus in lateral view (see Coronoid). Posteriorly, the surangular forms a lateral lip defining a posterodorsally facing surface that probably formed the anterolateral part of the area articularis mandibularis, as in most turtles. This part of the bone is poorly preserved. In *So. parsonsi* (TM 4023, possibly also JM SCHA 70), an anteroposteriorly directed ridge is present on the lateral surface of the surangular just anterior to the lateral lip contributing to the area articularis mandibularis. This ridge likely served for muscular attachment. There is apparently no such ridge in MJSN BEB011-13 (Fig. 6).

#### Coronoid

The coronoid contacts the dentary anterolaterally and anteromedially, the splenial ventromedially, the prearticular posteromedially, and the surangular posterolaterally (Fig. 6). As in *Solnhofia parsonsi* (TM 4023, JM SCHA 70) and sandownids (Evers & Joyce, 2020), the coronoid forms the posteromedial part of the triturating surface. Posteriorly, the coronoid forms the anterior wall of the fossa meckelii. In *So. parsonsi* (TM 4023), the posteroventral process of the coronoid on the medial side forms a large part of the medial wall of the fossa meckelii. The state of preservation of MJSN BEB011-13 prevents us from checking whether this is also the case in *So. brachyrhyncha*. The anterodorsal process of the surangular hides the main body of the coronoid in lateral view leaving only the coronoid process visible (see Surangular). In *So. parsonsi* (TM 4023), the surangular does not reach as high and the body of the coronoid is exposed laterally posteroventral to the processus coronoideus. As in many thalassochelydians (Anquetin, Püntener & Billon-Bruyat, 2015) and possibly *Sandownia harrisi* (Evers & Joyce, 2020), the medial surface of the coronoid is pierced by an unnamed foramen (Figs. 6G and 6H). As in *So. parsonsi* (TM 4023), the coronoid foramen lies close to the suture with the splenial. In contrast to *So. parsonsi* (TM 4023), the coronoid does not contribute to the formation of the foramen intermandibularis medius in *So. brachyrhyncha*. As in thalassochelydians and sandownids (Evers & Benson, 2019), the coronoid process is greatly developed and recurved posteriorly. The processus coronoideus of *So. brachyrhyncha* is somewhat stouter and less high than in *So. parsonsi* (TM 4023). The upper part of the processus coronoideus is striated.

#### Articular

Some of the right articular is preserved, but much of its dorsal aspect is eroded and encrusted by iron oxides (Fig. 6). The articular contacts the surangular anterolaterally, as well as the prearticular anterodorsally and the angular anteroventrally on the medial surface of the ramus. Most of these sutures are difficult to follow. Nothing meaningful can be said about the articular surface. The morphology of the articular is also little known in *Solnhofia parsonsi* (TM 4023, JM SCHA 70).

#### Prearticular

The prearticular contacts the coronoid anterodorsally, the splenial anteriorly, the angular ventrally, and the articular posteriorly (Figs. 6G and 6H). The sutures with the coronoid, splenial and articular are difficult to see. The prearticular probably contributes to the medial wall of the fossa meckelii, but the degree of contribution of the coronoid to this wall is uncertain in *Solnhofia brachyrhyncha* (see Coronoid). Ventrally, the prearticular forms the dorsal half of the foramen intermandibularis caudalis (see Angular). Posterodorsally, the prearticular forms a medial lip that was covered dorsally by the articular during life.

#### Splenial

As in *Solnhofia parsonsi* (TM 4023), the splenial is a long element covering a large part of the medial surface of the mandibular ramus and forming the medial wall of the sulcus cartilaginis meckelii (Figs. 6G and 6H). The splenial contacts the coronoid dorsally, the dentary anterodorsally and ventrally, the angular posteroventrally, and the prearticular posteriorly. In *So. parsonsi* (TM 4023), the splenial does not contact the dentary anterodorsally because the coronoid enters the margin of the foramen intermandibularis medius. In both *So. parsonsi* (TM 4023) and *So. brachyrhyncha*, the splenial extends anteriorly almost to the mandibular symphysis making the medially open part of the sulcus cartilaginis meckelii very short. The splenial is proportionally about the same size in the two species, but as the symphysis is much longer in *So. parsonsi* (TM 4023) the splenial is located more posteriorly along the mandibular ramus in that species. As a result, the splenial reaches posteriorly to the level of the middle of the dorsal opening of the fossa meckelii in *So. parsonsi* (TM 4023). In contrast, the splenial only reaches posteriorly to the level of the anteriormost point of the dorsal opening of the fossa meckelii in *So. brachyrhyncha*. This difference in the position of the splenial along the mandibular ramus in the two species is also apparent in the relative length of the ventral contacts of the splenial with the dentary and the angular. The contacts are about equal in length in *So. parsonsi* (TM 4023), whereas the anteroventral contact with the dentary is clearly longer than the posteroventral one with the angular in *So. brachyrhyncha* (Figs. 6G and 6H).

### Shell

#### General description

In addition to the cranium (MJSN BAN001-2.1) described above, the assemblage MJSN BAN001-2 is composed of over 180 shell bones in different preservation states: three neurals, ∼100 costals, 38 peripherals, ∼30 hyo- or hypolastra, eight xiphiplastra, as well as numerous unidentifiable shell fragments. Despite the presence of many sub-complete shell elements, only two corresponding pairs could be assembled (two hypoplastra and two xiphiplastra). The two hypoplastra that can be paired (MJSN BAN001-2.29; see Table 1) are assigned to a juvenile “plesiochelyid” (*Tropidemys*? *langii*?). These elements are described separately below. The specific assignment of many shell elements in the assemblage MJSN BAN001-2 remains uncertain. This concerns particularly the three neurals, parts of the costals and peripherals, all xiphiplastra, as well as all unidentifiable fragments. Although this may be criticized, we have decided to describe these elements alongside the ones we confidently refer to *Solnhofia brachyrhyncha*, but to conservatively assign them to Thalassochelydia indet. This is because we consider that most of these elements probably belong to the new species even though we cannot confirm it for the moment. Only the 27 best preserved shell elements are confidently referred to *Solnhofia brachyrhyncha* herein (see Table 1 and Discussion). They represent at least five different individuals (see Discussion). Their corresponding estimated carapace length varies from about 150 mm to about 250 mm. In comparison, the only specimen of *Solnhofia parsonsi* with a preserved shell (JM SCHA 70) has a carapace length of 184 mm (Joyce, 2000).

#### Neurals

**Figure 7:**
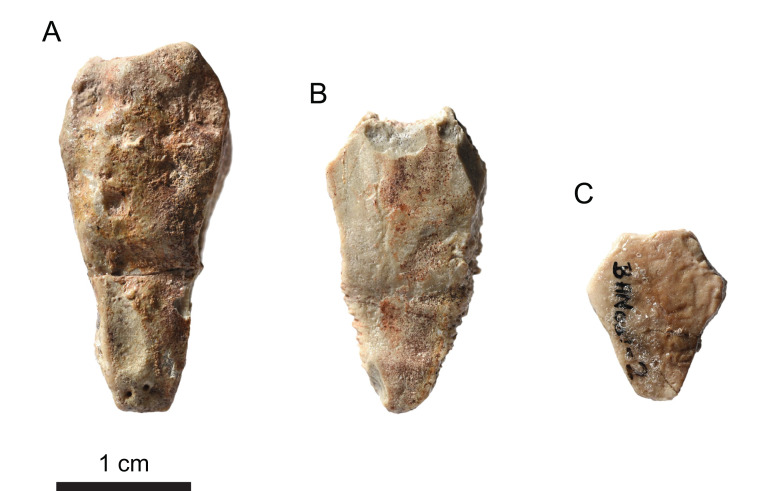
MJSN BAN001-2, Thalassochelydia indet. (Kimmeridgian, Porrentruy, Switzerland). (A–C) Neurals in dorsal view.

Only three neurals could be identified in the assemblage (Fig. 7). The neurals are elongated and hexagonal in outline, but differ strongly in length-width proportion and bone thickness. The proportionally longest neural (28 mm long; Fig. 7A) is 8 mm thick. Its relative length and a supposed mark of a crossing sulcus suggests that it is either a neural 3 or 5. The proportionally second longest neural (23 mm long; Fig. 7B) has a bone thickness of 5 mm, and the proportionally shortest neural (13 mm long; Fig. 7C) a bone thickness of 4 mm. The shape of the latter neural (relatively broad with more equally sized lateral sides) reminds the posterior neurals (i.e., neurals 6–8) of other thalassochelydian turtles. None of the three neurals can be confidently assigned to *Solnhofia brachyrhyncha* because there is a strong intra- and interspecific variation of these bones in thalassochelydian turtles. The neurals of *Solnhofia parsonsi* (JM SCHA 70) are generally wider than the three neurals found in the assemblage MJSN BAN001-2 (Joyce, 2000), especially compared to the proportionally longest one (Fig. 7A).

#### Costals

**Figure 8:**
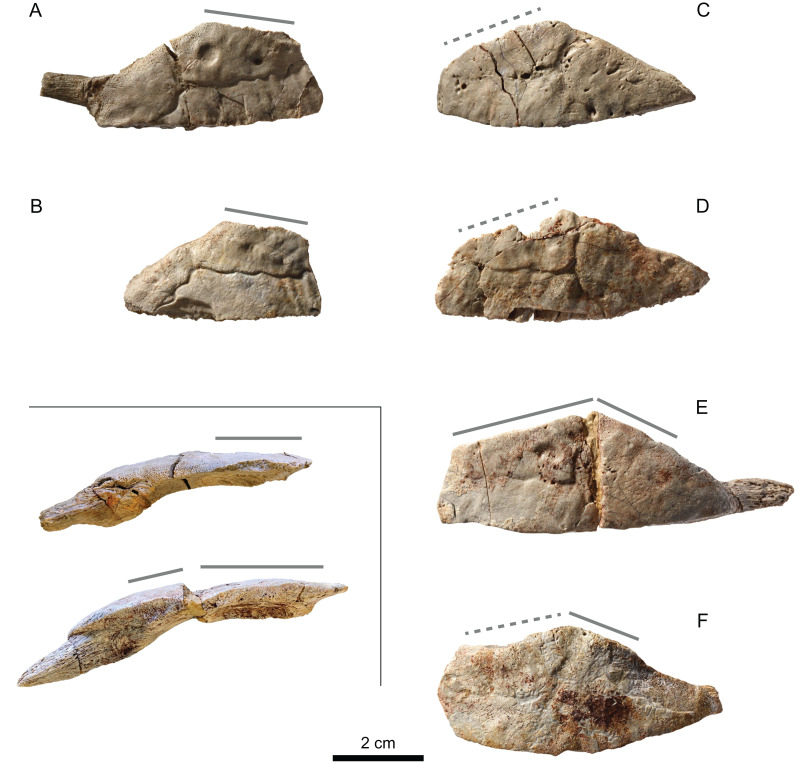
MJSN BAN001-2.2 to MJSN BAN001-2.7, paratypes of *Solnhofia brachyrhyncha* (Kimmeridgian, Porrentruy, Switzerland). (A) MJSN BAN001-2.2, left costal 1; (B) MJSN BAN001-2.3, left costal 1; (C) MJSN BAN001-2.4, right costal 1; (D) MJSN BAN001-2.5, right costal 1; (E) MJSN BAN001-2.6, right costal 1; (F) MJSN BAN001-2.7, right costal 1. Inset: top same as in (A) (mirrored for comparison) and bottom same as in (E) both in anterior view. Gray lines indicate sutural contacts with the nuchal (anteromedially) and first peripheral (anterolaterally; when present). All in dorsal view, except inset.

**Figure 9:**
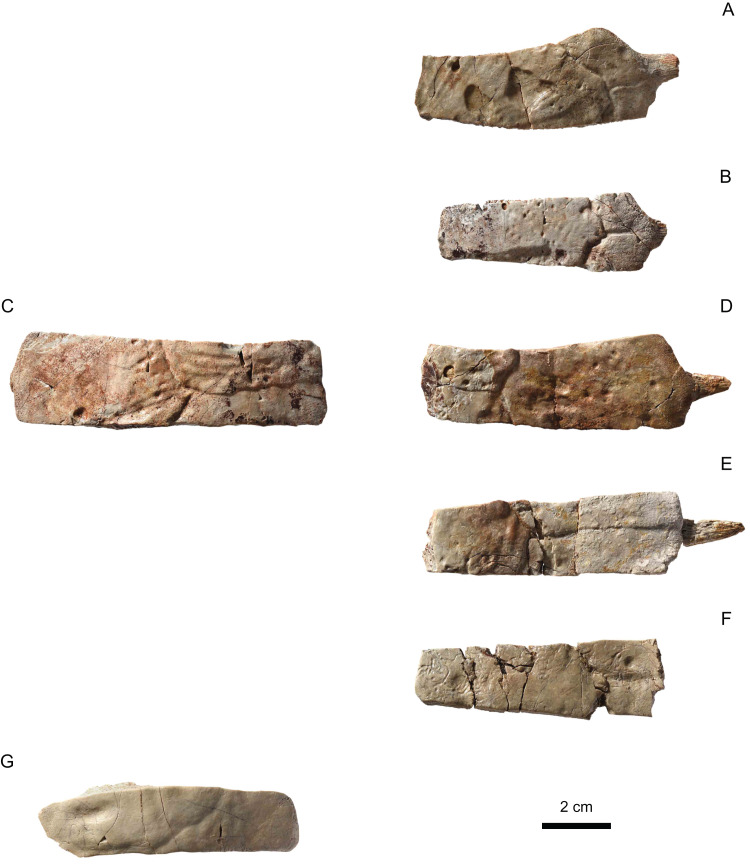
MJSN BAN001-2, Thalassochelydia indet. (Kimmeridgian, Porrentruy, Switzerland). (A and B) right costals 2; (C and D) left and right costals 3; (E and F) right costals 4; (G) left costal 6. All in dorsal view.

**Figure 10:**
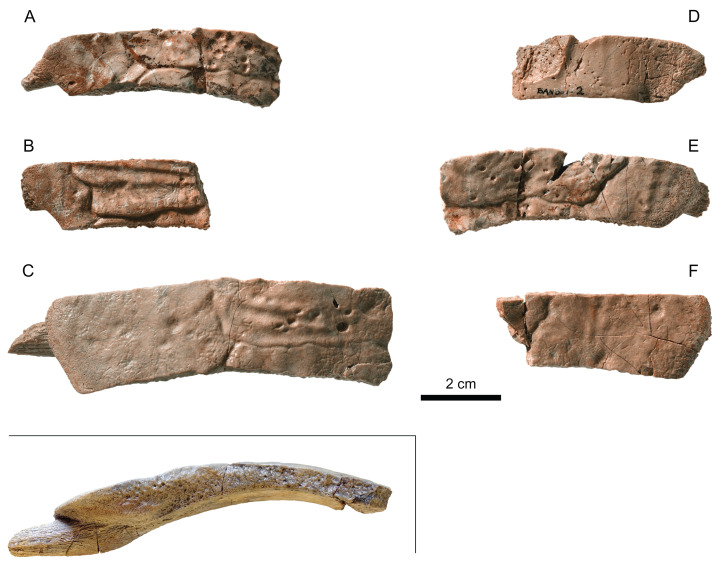
MJSN BAN001-2.8 to MJSN BAN001-2.13, paratypes of *Solnhofia brachyrhyncha* (Kimmeridgian, Porrentruy, Switzerland). (A) MJSN BAN001-2.8, left costal 5; (B) MJSN BAN001-2.9, left costal 5; (C) MJSN BAN001-2.10, left costal 5; (D) MJSN BAN001-2.11, right costal 5; (E) MJSN BAN001-2.12, right costal 5; (F) MJSN BAN001-2.13, right costal 5. Inset: same as in (C) in posterior view to document the distal thickening. All in dorsal view, except inset.

There are about 100 costals or parts of costals in the assemblage MJSN BAN001-2. None of these elements could be assembled together. All sufficiently preserved costals show a mainly non-sutural distal border indicating that costo-peripheral fontanelles were present. Some of the costals still possess laterally projecting ribs that are not covered by dermal bone. Costals 1 and 5 are the most frequently preserved costals in the assemblage (i.e., ten costals 1 and eleven costals 5), which is possibly due to their stronger build related to the attachment of the axillary and inguinal buttresses. Smaller costals 1 have a completely non-sutural lateral border (Figs. 8A–8D), but two relatively large costals 1 show a partially sutured contact with the first peripheral (Figs. 8E–8F). Ventrally, costal 1 has an articulation site for the first thoracic rib. The axillary buttress probably contacted the anterior surface of the distal part of the second thoracic rib (Fig. 8). Costals 2–5 have a completely non-sutural lateral border (Figs. 9A–9F and 10). The condition in costal 6 is not clear, as the only two preserved elements are incomplete laterally (Fig. 9G). The inguinal buttress is indicated by a strong lateral thickening of costals 5 and 6 (only the anterior border of the latter), but it is uncertain whether or not the buttress contacted the costal bones as no articulation scar is visible (Fig. 10). No costals 7–8, pygals and suprapygals could be identified.

**Figure 11:**
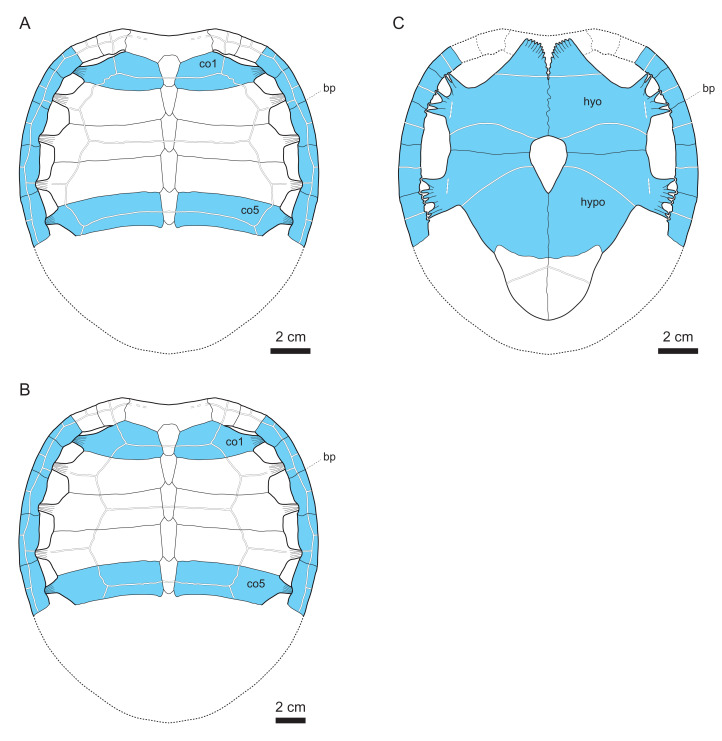
Tentative reconstruction of the shell of *Solnhofia brachyrhyncha* based on isolated bones from the assemblage MJSN BAN001-2. Partial reconstruction of the carapace in an early (A) and a more advanced (B) ontogenetic stage; (C) reconstruction of the plastron. In blue, the elements of MJSN BAN001-2 that could be attributed to *Solnhofia brachyrhyncha*. Thick lines: natural borders; thin lines: bone sutures; double lines: scute sulci. Abbreviations: bp, bridge peripherals; co, costal; hyo, hyoplastron; hypo, hypoplastron.

The width of the vertebral scutes varies considerably between the different costals. The vertebrals covered almost the entire costal in relatively small specimens (e.g., Figs. 8B, 9B and 11A), but only about half of the costal width in the largest preserved costal 5 (Figs. 10C and 11B). However, vertebral width can also vary between similarly sized specimens, indicating that there must be other factors besides age controlling this feature (if all of these specimens truly belong to a single species). A medium sized costal 6 shows a strong widening of the anterolateral margin of vertrebral 4 (Fig. 9G), a feature that can also notably be observed in *Plesiochelys etalloni* and *Plesiochelys bigleri* (Anquetin, Püntener & Billon-Bruyat, 2014; Püntener, Anquetin & Billon-Bruyat, 2017a).

Only the better preserved costals 1 (MJSN BAN001-2.2 to MJSN BAN001-2.7; Fig. 8; Table 1) and costals 5 (MSJN BAN001-2.8 to MJSN BAN001-2.13; Fig. 10; Table 1) are herein assigned to *Solnhofia brachyrhyncha*, notably based on their non-sutural lateral border and general resemblance with those of *So. parsonsi* (see Discussion). Comparing similarly sized specimens, the costo-peripheral fontanelles of *So. parsonsi* (JM SCHA 70) are not as much developed anteriorly and posteriorly (Joyce, 2000). Costal 1 is completely sutured to the first two peripherals in *So. parsonsi* (JM SCHA 70), whereas the larger specimens referred to *So. brachyrhyncha* only show a partial sutural contact between costal 1 and peripheral 1 (Figs. 8E–8F and 11B). Similarly, costal 5 is partially sutured to the peripherals in *So. parsonsi* (JM SCHA 70), but costal 5 has a completely non-sutural lateral border in *So. brachyrhyncha* (Figs. 10 and 11A–11B). The ribs of costals 1 and 5 are significantly thickened distally in relation with the nearby presence of the axillary and inguinal buttresses (Figs. 8 and 10). Although a contact between the buttress and the rib tip is apparently present in costal 1 and cannot be excluded in costal 5, it is important to note that the ventral aspect of these costals does not bear any scar for the attachment of the axillary and inguinal buttresses. The vertebral scutes of *So. parsonsi* (JM SCHA 70) are approximately as wide as in similarly sized specimens of *So. brachyrhyncha* (Fig. 11B).

#### Peripherals

**Figure 12:**
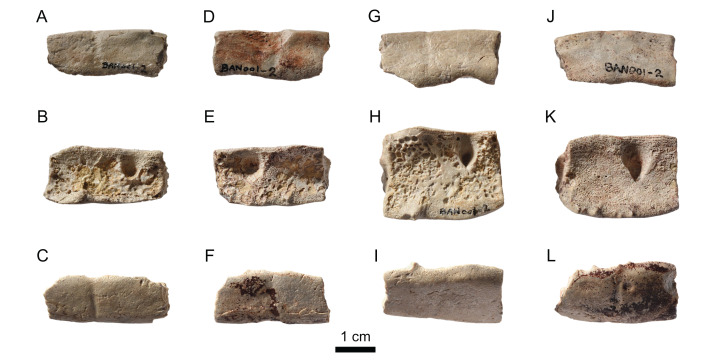
MJSN BAN001-2.14 to MJSN BAN001-2.17, paratypes of *Solnhofia brachyrhyncha* (Kimmeridgian, Porrentruy, Switzerland). (A–C) MJSN BAN001-2.14, bridge peripheral; (D–F) MJSN BAN001-2.15, bridge peripheral; (G–I) MJSN BAN001-2.16, bridge peripheral; (J–L) MJSN BAN001-2.17, bridge peripheral. Each peripheral is represented in dorsal (A, D, G and J), visceral (B, E, H and K) and ventral (C, F, I and L) views.

Thirty-eight peripherals were found in the assemblage MJSN BAN001-2. Peripherals of the bridge region are the best preserved elements (Fig. 12). They consist in wedge-shaped, elongate and relatively narrow elements. The dorsomedial and ventromedial edges of the latter show a non-sutural contact to the rest of the carapace and the plastron respectively, as common in “eurysternids” The dorsomedial edge has a shallow, roundish notch just above the rib socket (Figs. 12A, 12D, 12G and 12J). The ventromedial edge of some of the bridge peripherals exhibits small protuberances that serve as loose articulation sites for the short peg-like projections of the hyo- and hypoplastra (Figs. 11C, 12I and 12L), similar to the condition in *Solnhofia parsonsi* (JM SCHA 70). The sockets in which the peripherals receive the costal ribs are somewhat teardrop-shaped, with the cusp pointing ventrally (best visible in Figs. 12H and 12K). In contrast, these sockets are roundish to oval-shaped and situated more dorsally in juvenile specimens of *Plesiochelys bigleri* (MJSN SCR010-327). Intermarginal scute sulci are apparent in some peripherals, but other sulci are difficult to discern. The four best preserved bridge peripherals (MJSN BAN001-2.14 to MJSN BAN001-2.17; Fig. 12) are herein assigned to *Solnhofia brachyrhyncha*. Their length-width proportions are similar to *So. parsonsi* (JM SCHA 70). Very wide posterior peripherals such as the one occurring in *So. parsonsi* (JM SCHA 70) could not be found in the assemblage MJSN BAN001-2.

#### Hyo- and hypoplastra

**Figure 13:**
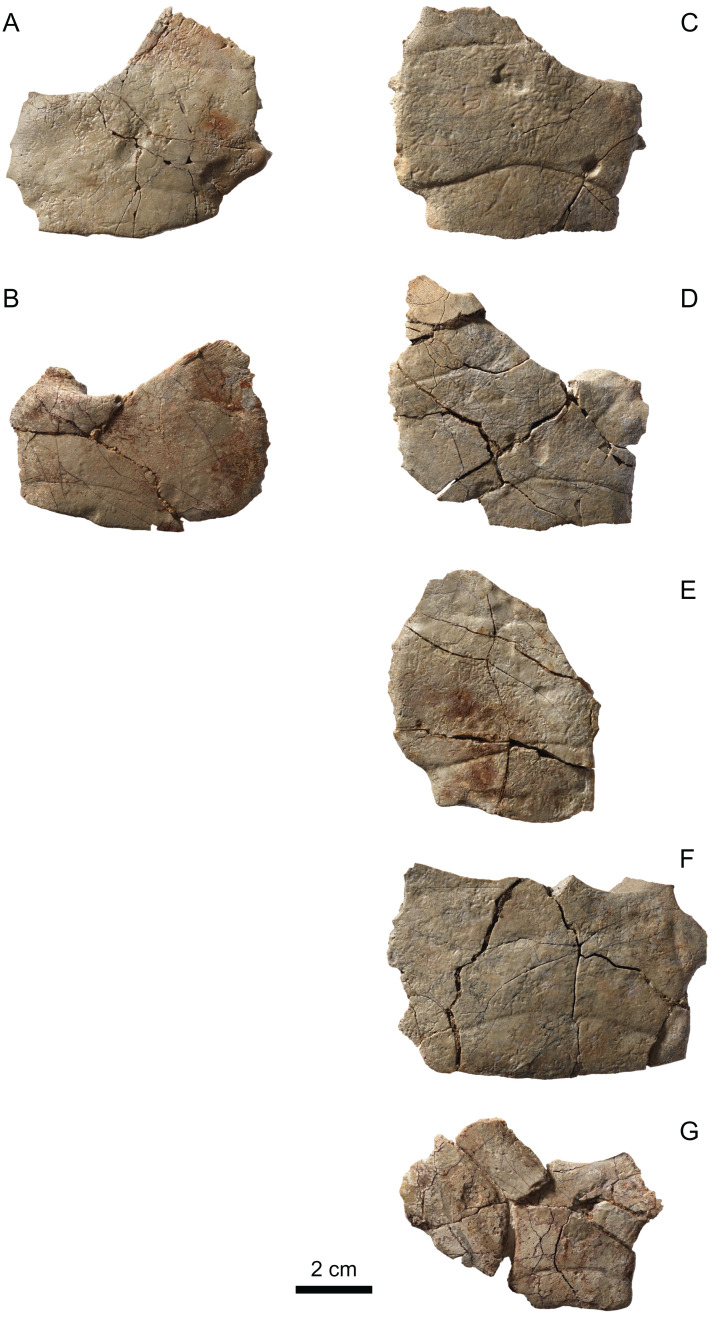
MJSN BAN001-2.18 to MJSN BAN001-2.24, paratypes of *Solnhofia brachyrhyncha* (Kimmeridgian, Porrentruy, Switzerland). (A) MJSN BAN001-2.18, right hyoplastron; (B) MJSN BAN001-2.19, right hyoplastron; (C) MJSN BAN001-2.20, left hyoplastron; (D) MJSN BAN001-2.21, left hyoplastron; (E) MJSN BAN001-2.22, left hyoplastron; (F) MJSN BAN001-2.23, left hyoplastron; (G) MJSN BAN001-2.24, left hyoplastron. All in ventral view.

**Figure 14:**
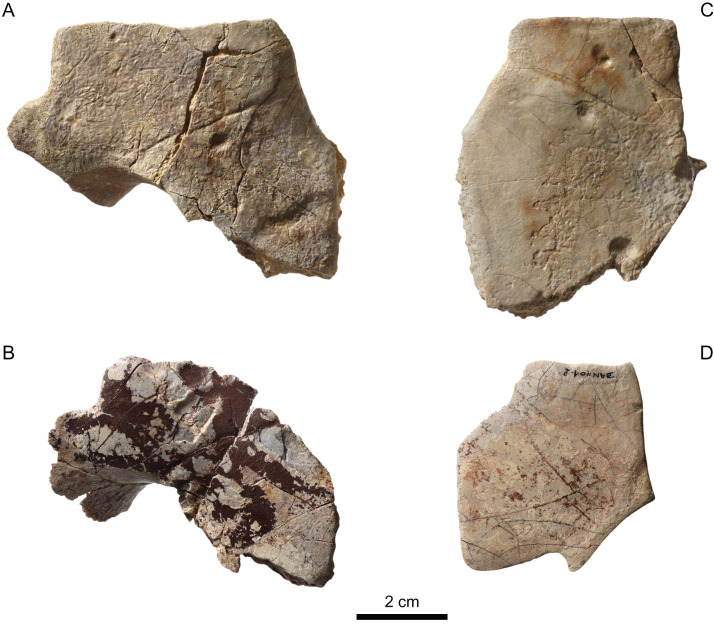
MJSN BAN001-2.25 to MJSN BAN001-2.28, paratypes of *Solnhofia brachyrhyncha* (Kimmeridgian, Porrentruy, Switzerland). (A) MJSN BAN001-2.25, right hypoplastron; (B) MJSN BAN001-2.26, right hypoplastron; (C) MJSN BAN001-2.27, left hypoplastron; (D) MJSN BAN001-2.28, left hypoplastron. All in ventral view.

There are about 30 hyo- or hypoplastral elements in the assemblage MJSN BAN001-2. Only thirteen of these could be identified. Seven hyoplastra (MJSN BAN001-2.18 to MJSN BAN001-2.24; Fig. 13) and four hypoplastra (MJSN BAN001-2.25 to MJSN BAN001-2.28; Fig. 14) are assigned to *Solnhofia brachyrhyncha*, while two hypoplastra are referred to a juvenile “plesiochelyid” specimen (see below). The plastron of *So. brachyrhyncha* is characterised by the following features: presence of central and lateral plastral fontanelles, a ligamentous bridge, and a non-sutural contact between the hyoplastra and the anterior plastral elements (Fig. 11C). The central plastral fontanelle is somewhat escutcheon shaped, that is, broadly roundish to quadrangular between the hyoplastra and tappering posteriorly between the hypoplastra (Figs. 11C, 13 and 14). The smaller part of it is situated between the hyoplastra, the better part being comprised between the hypoplastra. In contrast, the (slightly bigger) central plastral fontanelle of *So. parsonsi* (JM SCHA 70) is oval in outline and situated more anteriorly (the hyoplastral part being about as long as the hypoplastral part). The lateral plastral fontanelles are somewhat rectangular in outline, and reach more anteriorly than the central plastral fontanelle. They may have been slightly longer than in *So. parsonsi* (JM SCHA 70).

The anteromedial border of the hyoplastra is never completely preserved, but one left hyoplastron (MJSN BAN001-2.21; Fig. 13D) shows a rudimentary row of small projections towards the (missing) ento- and epiplastron. This suggests that there was no sutural connection between the two latter bones and the hyoplastron. As in *So. parsonsi* (JM SCHA 70), the hyoplastron appears to be about as wide as long. The lateral border of the hyoplastra presents distinct peg-like projections towards the peripherals (Figs. 11C, 13D and 13F), while in *So. parsonsi* (JM SCHA 70) these projections are mostly fused together to form a continuous plate. The lateral border of the hypoplastra is less well preserved (Fig. 14). The axillary notch of *So. brachyrhyncha* appears to be more open medially than in *So. parsonsi* (e.g., Figs. 12A and 12G). However, it should be noted that postmortem deformation can affect the shape of the axillary notch. The posterior border of the hyoplastra is always slightly convex, and the anterior border of the hypoplastra correspondingly concave.

The posterior sulcus of the humeral scute is straight and situated anterior to the deepest point of the axillary notch (Fig. 11C). The sulcus between the pectoral and abdominal scutes runs approximately at the same level as the anterior end of the central plastral fontanelle. This sulcus is always curved towards the anterior, which contrasts to the straight sulcus observed in *So. parsonsi* (JM SCHA 70). However, based on our own experience with the plesiochelyid *Plesiochelys bigleri* (Püntener, Anquetin & Billon-Bruyat, 2017a, 2017b), we know that the shape of this sulcus can change intraspecifically in some individuals. So until more material of *So. parsonsi* is known, it is better to avoid using such differences for systematic purposes. The abdominal-femoral sulcus extends from the inguinal notch and almost reach the middle of the central plastral fontanelle anteromedially. Only a few traces of the medial sulcus of the inframarginal scutes can be discerned.

#### Xiphiplastra

**Figure 15:**
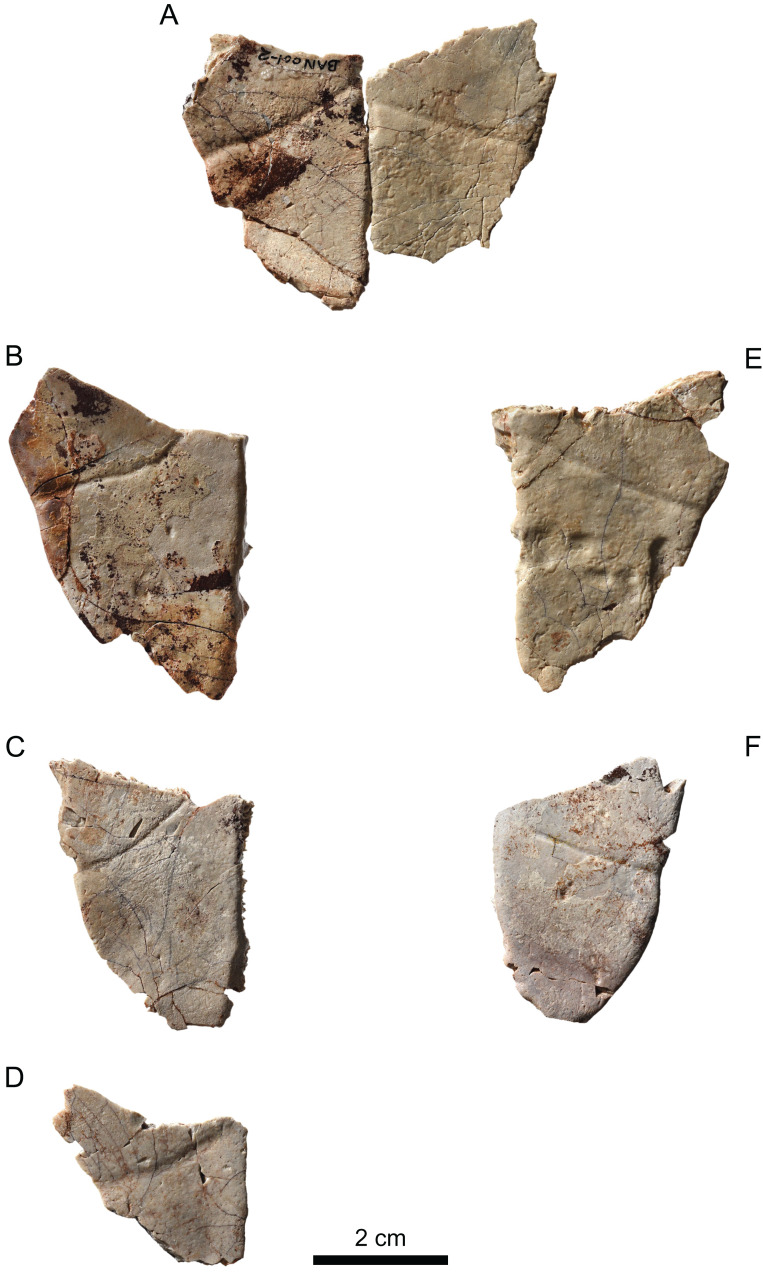
MJSN BAN001-2, Thalassochelydia indet. (Kimmeridgian, Porrentruy, Switzerland). (A) left and right xiphiplastra of the same individual; (B–D), right xiphiplastra; (E and F) left xiphiplastra. All in ventral view.

Four right and three left xiphiplastra are partially preserved (Fig. 15). One pair could be assembled (Fig. 15A). The preserved xiphiplastra are triangular in shape and longer than wide. There is no sign of a xiphiplastral fontanelle in any of them. The sulcus between the femoral and anal scutes is restricted to the xiphiplastra in the two assembled bones, but seems to extend to the hypoplastron in other specimens of the assemblage MJSN BAN001-2 (e.g., Figs. 15B and 15C). The condition in *Solnhofia parsonsi* (JM SCHA 70) is uncertain (Joyce, 2000). But it should be noted that this feature is also a common intraspecific variation in “plesiochelyids” (e.g., *Plesiochelys bigleri* and *Plesiochelys etalloni*; Püntener, Anquetin & Billon-Bruyat, 2017a). None of the xiphiplastra can be confidently assigned to *Solnhofia brachyrhyncha*. In contrast to the relatively long xiphiplastra found in the assemblage MJSN BAN001-2, this element is about as long as wide in *So. parsonsi* (JM SCHA 70).

## Description of MJSN BAN001-2.29 (*Tropidemys*? *langii*?)

**Figure 16:**
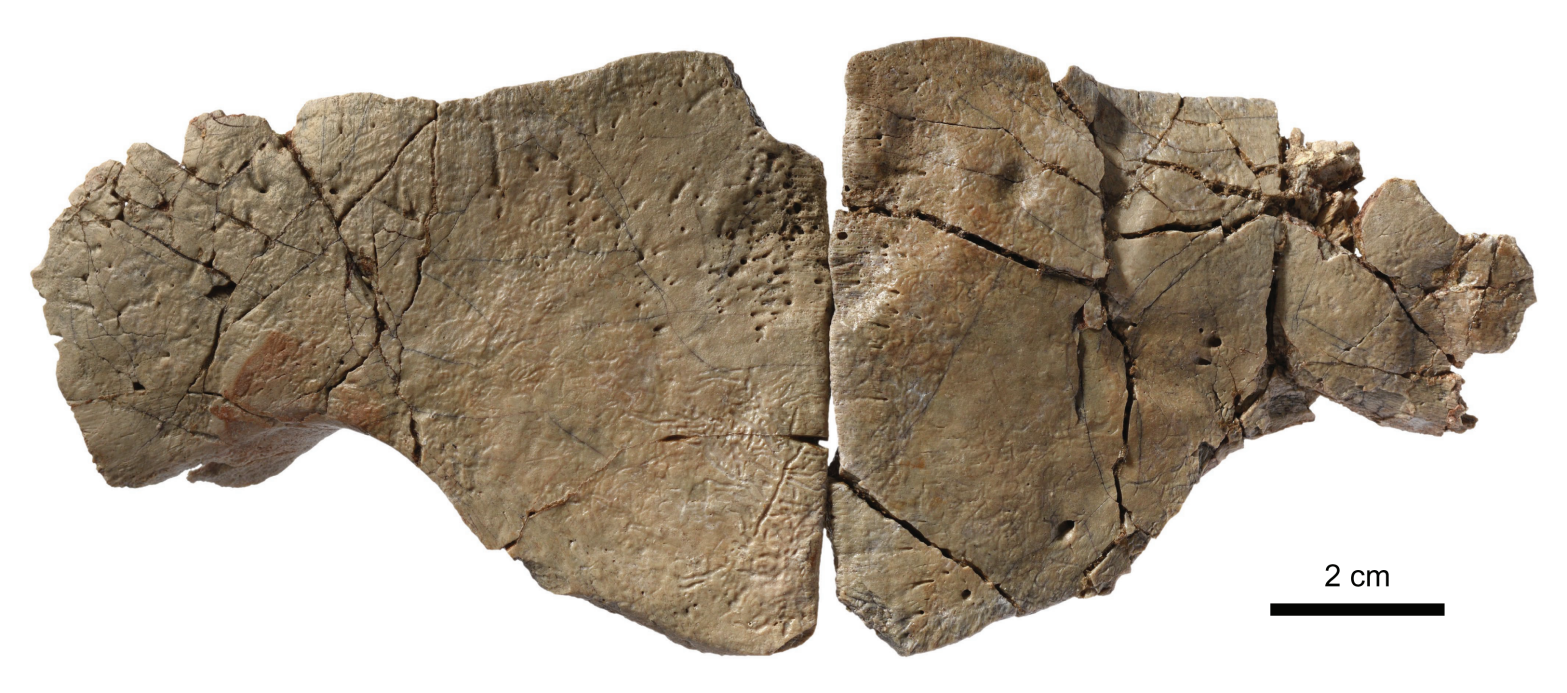
MJSN BAN001-2.29, “Plesiochelyidae”, *Tropidemys*? *langii*? (Kimmeridgian, Porrentruy, Switzerland). Left and right hypoplastra of the same individual, in ventral view.

In addition to the material described above, two elements of the assemblage MJSN BAN001-2 are assigned to a juvenile plesiochelyid turtle. They are probably the left and right hypoplastra of the same specimen, which had an estimated carapace length of about 250 mm (Fig. 16). There is clearly no central fontanelle, and prominent lateral fontanelles, as in *Solnhofia brachyrhyncha*, can be excluded. Although the lateral borders are incomplete, their preservation is sufficient to exclude peg-like projections to the peripherals and therefore suggest an osseous bridge. The relatively wide bridge reminds the condition in adult specimens of *Tropidemys langii* (see Discussion). Scute sulci cannot be discerned.

## Systematic Paleontology

**TESTUDINATA** Klein, 1760

**THALASSOCHELYDIA** Anquetin, Püntener & Joyce, 2017

***Solnhofia*** Gaffney, 1975

**Type species.** *Solnhofia parsonsi* Gaffney, 1975.

**Included valid species.** *Solnhofia parsonsi* Gaffney, 1975; *Solnhofia brachyrhyncha* n. sp.

**Occurrence.** Early and late Kimmeridgian of Porrentruy, Canton of Jura, Switzerland (this study); late Kimmeridgian of Solothurn, Canton of Solothurn, Switzerland (Gaffney, 1975); Kimmeridgian of Labastide-Murat, Lot, France (Lapparent de Broin, Lange-Badré & Dutrieux, 1996); Kimmeridgian/Tithonian and early Tithonian of Schamhaupten and Solnhofen, Bavaria, Germany (Parsons & Williams, 1961; Gaffney, 1975; Joyce, 2000); Tithonian of Canjuers, Var, France (Broin, 1994).

**Revised diagnosis.** *Solnhofia* differs from all other “eurysternids” sensu Anquetin, Püntener & Joyce (2017) by having an enlarged skull (40% of carapace length in *So. parsonsi*, at least more than 25% in *So. brachyrhyncha*), a complete secondary palate formed primarily by the maxilla, limited temporal emargination, a smooth upper triturating surface without lingual ridge, a reduced foramen palatinum posterius, a massive processus trochlearis oticum that obscures the foramen nervi trigemini in lateral view, a low to non-existent labial ridge on the mandible, reduced costo-peripheral fontanelles, wide vertebral scutes, hyoplastra about as wide as long, lateral plastral fontanelles, and a central plastral fontanelle.

*Solnhofia* differs from sandownids sensu Evers & Benson (2019) by having a less extensively developed secondary palate formed mostly by the maxilla and premaxilla with only small or no contribution from the vomer and palatine, an acute and high labial ridge on the maxilla, a jugal medial process limited to the dorsal surface of the maxilla and pterygoid (no contribution of the jugal to the palatal surface; possibly also the case in *Leyvachelys cipadi*), a longer than tall maxilla (as opposed to very tall and short in sandownids), a fossa orbitalis level with the fossa nasalis (consistently above the fossa nasalis in sandownids), a processus pterygoideus externus (albeit reduced), a processus trochlearis oticum limited to the medial part of the otic chamber, a well developed contribution of the parietal to the formation of the processus trochlearis oticum, an open incisura columellae auris (also in *Brachyopsemys tingitana*), a more pronounced infolding ridge on the posterior surface of the quadrate, and finally by lacking a posteromedial extension of the pterygoid covering most of the parabasisphenoid and basioccipital in ventral view. In addition, *Solnhofia* differs from *Leyvachelys cipadi* (the only sandownid known from the shell) by having costo-peripheral fontanelles, a ligamentous bridge, and lateral plastal fontanelles.

**Remarks.** Some of the characteristics listed above to differentiate *Solnhofia* from “eurysternids” are shared by sandownids (e.g., presence of a secondary palate, reduced temporal emargination, reduced foramen palatinum posterius, massive processus trochlearis oticum; Evers & Joyce, 2020). Waiting for a reevaluation of the phylogenetic position of *Solnhofia* relative to Thalassochelydia and Sandownidae (see Introduction), it is unclear whether these characteristics are shared derived features of *Solnhofia* + Sandownidae outside Thalassochelydia, or of *Solnhofia* + Sandownidae within Thalassochelydia, or morphological convergences between *Solnhofia* (a thalassochelydian) and Sandownidae notably related to the presence of an extensive secondary palate. Instinctively, we would favor the hypothesis where sandownids are derived thalassochelydians closely related to *Solnhofia*, but this would require a rigorous testing that goes beyond the purpose of the present study. For the time being, we conservatively refer *Solnhofia* to Thalassochelydia herein, but include comparisons with Sandownidae in the revised diagnosis.

***Solnhofia brachyrhyncha* n. sp.**

urn:lsid:zoobank.org:act:F6C29ABB-1CCC-4E91-8653-95AE1691CF4E

Figs. 3–6, 8 and 10–14

**Etymology.** The species name is derived from the Greek “*brachys”* meaning short and “*rhynchos*” meaning snout, beak.

**Holotype.** MJSN BAN001-2.1, a relatively completed, but crushed cranium (Figs. 3 and 4).

**Paratypes.** MJSN BAN001-2.2 to MJSN BAN001-2.28, twelve costals, four peripherals, seven hyoplastra, and four hypoplastra found associated with the holotype, but representing at least five different individuals (Figs. 8, 10 and 14).

**Type horizon and locality.** “Le Banné” (BAN), a hill southwest of Porrentruy, Canton of Jura, Switzerland. Banné Marls, Banne Member, Reuchenette Formation, early Kimmeridgian, Late Jurassic (Comment, Ayer & Becker, 2011; Comment et al., 2015).

**Referred specimens.** MJSN SCR010-1214, a severely crushed and eroded cranium (Fig. 5); MJSN BEB011-13, a small mandible missing the posterior part of the left ramus (Fig. 6).

**Additional occurrence.** Early and late Kimmeridgian of Courtedoux, Canton of Jura, Switzerland.

**Diagnosis.** *Solnhofia brachyrhyncha* is diagnosed as a representative of *Solnhofia* by the full list of characteristics provided for this taxon above. *Solnhofia brachyrhyncha* differs from *Solnhofia parsonsi* by having a shorter and broader cranium (only slightly longer than wide), a shorter upper triturating surface that is wider posteriorly with a slightly sinusoidal labial margin, a palatine that does not contribute to the triturating surface nor partially floors the narial passage, a processus inferior parietalis that forms most of the anterior and posterior margin of the foramen nervi trigemini and that has a broader contact with the pterygoid behind that foramen, a reduced lateral exposure of the epipterygoid without blunt process, a processus trochlearis oticum more oblique in dorsal or ventral view and less concave in anterior view, a reduced contribution of the prootic to the processus trochlearis oticum, choanae limited to the palatines, a more developed processus pterygoideus externus, a condylus mandibularis situated anterior to the level of the occipital plane, a longer ventral exposure of the parabasisphenoid, a mandible about as wide as long with a short symphysis, a large laterally projecting dentary tubercle, a shorter lower triturating surface that widens greatly posteriorly and has a concave labial margin, a stouter and shorter coronoid process with a striated upper part, a greater anterodorsal development of the surangular (hidding much of the coronoid body in lateral view), a splenial positioned more anteriorly along the mandibular ramus, a foramen intermandibularis medius formed only by the dentary and splenial (no contribution from the coronoid), costo-peripheral fontanelles extending more anteriorly (to costal 1) and posteriorly (beyond costal 5) along the costal series, and an escutcheon shaped central plastral fontanelle.

**Remarks.** MJSN BAN001-2 is an assemblage consisting of the holotype of *Solnhofia brachyrhyncha* and over 180 disarticulated shell bones (see Material above). Only part of these shell bones are here used to define this new species (paratypes). In this assemblage, two hypoplastra pertaining to a single individual clearly belong to a different taxon and are here tentatively interpreted to represent a juvenile specimen of the thalassochelydian *Tropidemys langii* (see below). The remaining shell bones in this assemblage are conservatively identified as Thalassochelydia indet.

**“PLESIOCHELYIDAE”** Baur, 1888 sensu Anquetin, Püntener & Joyce (2017)

***Tropidemys*? *langii*?** Rütimeyer, 1873

Fig. 16

**Referred material.** MJSN BAN001-2.29, a left and a right hypoplastra probably belonging to the same individual found in association with the material referred to the new species *Solnhofia brachyrhyncha* (see above).

## Discussion

### Assignment of shell elements

As noted above and discussed below, the assemblage MJSN BAN001-2 is composed of over 180 disarticulated shell bones. Many of these elements have a “eurysternid” morphology (see below), but others are undiagnostic or too poorly preserved and at least one pair of hypoplastra can be confidently assigned to a “plesiochelyid” turtle. Therefore, the assemblage cannot be simply assumed to be monospecific, despite the fact that the morphology of most elements is relatively congruent and suggest the new species is largely dominant in the assemblage. It should also be noted that although *Solnhofia parsonsi* was recently proposed to be closely related to sandownids based on several cranial characters (Evers & Joyce, 2020), its shell morphology is more similar to that of “eurysternid” turtles (Joyce, 2000; Anquetin, Püntener & Joyce, 2017; Püntener, Anquetin & Billon-Bruyat, 2020). The shell of sandownids is only known in *Leyvachelys cipadi* and is notably characterized by strongly ossified costo-peripherals contacts (no fontanelles), a sutural bridge, and no lateral plastral fontanelles (Cadena, 2015). This explains why the following discussion mostly refers to the “eurysternid” shell morphology.

Based on their association with a cranium referable to *Solnhofia* and their resemblance to *So. parsonsi*, several shell elements can be confidently assigned to the new species *So. brachyrhyncha*. For a recent discussion about the shell anatomy of “eurysternids”, the reader is referred to Anquetin, Püntener & Joyce (2017) and Püntener, Anquetin & Billon-Bruyat (2020). *Solnhofia parsonsi* is notably characterized by a shell with small costo-peripheral fontanelles, a relatively well ossified plastron, a ligamentous bridge, semilunate lateral plastral fontanelles, an oval central plastral fontanelle, and a hyoplastron about as wide as long. Eleven of the about 30 hyo- and hypoplastra found in the assemblage match fairly well with this description and resemble those of *So. parsonsi* (MJSN BAN001-2.18 to MJSN BAN001-2.28), although some notable differences in the shape of the plastral fontanelles are present (see Description). They are referred to the new species herein.

A pair of hypoplastra (MJSN BAN001-2.29) lacking a central plastral fontanelle and suggesting an osseous bridge differs from the rest. Juvenile “plesiochelyid” specimens are rare, but we had access to comparative material for two species: *Plesiochelys bigleri* (MJSN SCR010-327 and MJSN BSY008-848) and *Plesiochelys etalloni* (NMS 9148). The two complete plastra of MJSN SCR010-327 and NMS 9148 are already fully ossified, as in the adults (no fontanelles, osseous bridge). A small lateral plastral fontanelle occurs only in a plastral fragment of MJSN BSY008-848, but the contact with the peripherals is sutural as in the other two specimens. These observations confirm that known plastral differences between “eurysternids” and “plesiochelyids” are already expressed in juvenile specimens. The pair of hypoplastra MJSN BAN001-2.29 is referred to a “plesiochelyid” turtle and reminds the condition observed in *Tropidemys langii* (see Description).

With about 100 elements, costals represent the most abundant shell bones in the assemblage MJSN BAN001-2. All sufficiently preserved costals are well ossified distally but show a non-sutural contact with peripherals suggesting the presence of small costo-peripheral fontanelles. The carapace of most “plesiochelyids” is completely ossified in the adults, as well as in the juveniles of some species (e.g., NMS 9148, *Plesiochelys etalloni*). However, in juveniles of *Plesiochelys bigleri* (MJSN BSY008-848 and MJSN SCR010-327) costo-peripheral fontanelles occur in costals 2 and 3, while costals 1 and 5–8 have sutural contacts with peripherals. The condition of costal 4 in these specimens is unknown. Therefore, we consider that costals 2–4 of the assemblage MJSN BAN001-2 cannot be confidently assigned to either “eurysternids” or juvenile “plesiochelyids”. In contrast, the twelve best preserved costals 1 and 5 (MJSN BAN001-2.2 to MJSN BAN001-2.13) match fairly well with similar elements in *So. parsonsi* and are consequently referred to the new species herein.

Peripherals are relatively poorly preserved in the assemblage MJSN BAN001-2, to the exception of some bridge peripherals. Their morphology (narrow, wedge-shaped elements with ligamentous dorsomedial and ventromedial contacts) indicate a shell with costo-peripheral fontanelles and a ligamentous bridge. As in *So. parsonsi*, some bridge peripherals bear small protuberances on their ventromedial edge receiving the short peg-like projections of the hyo- and hypoplastra. The best preserved bridges peripherals (MJSN BAN001-2.14 to MJSN BAN001-2.17) are therefore assigned to the new species herein.

Other elements, such as the neurals and the xiphiplastra, as well as the remaining costals, peripherals, hyo- and hypoplastra, are considered to be undiagnostic or too poorly preserved to warrant a safe taxonomic assignment. For the time being, they are conservatively referred to Thalassochelydia indet.

#### Solnhofia brachyrhyncha n. sp.

The description of the new species *Solnhofia brachyrhyncha* is primarily based on the cranium MSJN BAN001-2.1, which we herein designate as the holotype, and the incomplete skull MJSN SCR010-1214. These two skulls exhibit numerous morphological similarities with the different crania referred to *Solnhofia parsonsi*, justifying according to us the referral of the new species to the genus *Solnhofia*. The most striking of these similarities are: a relatively large head size relative to the length of the carapace; the presence of an extensive secondary palate formed primarily by the maxilla with an acute and moderately high labial ridge and a nonexistent lingual ridge; the presence of a greatly developed processus trochlearis oticum hiding the foramen nervi trigemini in lateral view; the contribution of both the parietal and quadratojugal to the formation of the processus trochlearis oticum; the presence of a large contact between the jugal and palatine in the floor of the fossa orbitalis; the presence of a well developed, ridge-like posteromedial process of the jugal running on the dorsal surface of the maxilla and pterygoid in the anteromedial margin of the fossa temporalis inferior. As noted recently by Evers & Joyce (2020), some of these features are also present in some sandownids and may indicate closer relationships of *Solnhofia* with sandownids than with thalassochelydians, or that sandownids are indeed derived thalassochelydians. However, these observations have not yet been tested in a phylogenetic context and until this is done we refrain from further comments on the matter. Furthermore, the present study indubitably shows that *So. parsonsi* is morphologically closer to *So. brachyrhyncha* than to any sandownids, so the debate about the phylogenetic position of sandownids has little incidence on our results.

Despite the obvious overall similarity, the cranium of *So. brachyrhyncha* differs from that of *So. parsonsi* in many aspects, the most important of which are discussed here. The cranium of *So. parsonsi* is much elongated, whereas it is only slightly longer than wide in *So. brachyrhyncha* (Table 2). The processus inferior parietalis forms most of the anterior and posterior margin of the foramen nervi trigemini and has a broader contact with the pterygoid posterior to that foramen (Fig. 4). In *So. parsonsi*, the pterygoid contributes more to the formation of the foramen nervi trigemini and its contact with the parietal posterior to the foramen is much shorter, so that the prootic comes much closer to the posterior margin of the foramen nervi trigemini. The epipterygoid is less exposed laterally anterior to the foramen nervi trigemini and has a smooth surface in *So. brachyrhyncha*, whereas the lateral exposure is greater and a blunt ventral process is present in *So. parsonsi* (Fig. 4). The triturating surface of *So. brachyrhyncha* is proportionally shorter and broader posteriorly (Fig. 3). Each ramus of the triturating surface is very wide posteriorly and narrows anteriorly, more so from the level of the middle of the orbit, which results in a slightly sinusoidal labial ridge. In contrast, the triturating surface of *So. parsonsi* is V-shaped (straight labial ridge) and elongated, and the width of each ramus is more or less constant. In contrast to *So. parsonsi*, the palatine does not contribute to the triturating surface and does not partially floor the narial passage in *So. brachyrhyncha* (Fig. 3).

The processus trochlearis oticum of *So. brachyrhyncha* is more oblique in dorsal or ventral view, less concave in anterior view, and has a smaller contribution of the prootic (Figs. 3–5). In *So. parsonsi*, the processus trochlearis oticum is more transverse in dorsal or ventral view, more concave in anterior view, and has a greater contribution of the prootic (Fig. 4). The choanae are limited to the palatines in *So. brachyrhyncha* (Fig. 3), whereas they extend posteriorly onto the pterygoids in *So. parsonsi*. The processus pterygoideus externus of *So. brachyrhyncha*, albeit reduced compared to most turtles, is more developed than in *So. parsonsi*, where it is reduced to a small nubbin (Figs. 3–4). The condylus mandibularis is situated anterior to the level of the occipital plane in *So. brachyrhyncha* (Fig. 3), while it is at the level of the occipital plane in *So. parsonsi*. Finally, the parabasisphenoid of *So. brachyrhyncha* has a greater ventral exposure and almost reaches the level of the posterior part of the processus pterygoideus externus anteriorly (Fig. 3), whereas it barely goes beyond the anterior margin of the pterygoid fossa in *So. parsonsi*.

The mandible of *So. brachyrhyncha* (MJSN BEB011-13) reminds of that of *So. parsonsi* in having a flat triturating surface with no labial or lingual ridge and a relatively low profile in lateral view. However, numerous characteristics separate the two species. The mandible of *So. brachyrhyncha* is almost as wide as long, whereas it is clearly longer than wide in *So. parsonsi* (Table 2). The symphysis is relatively short in *So. brachyrhyncha* and only partly covered by the triturating surface dorsally (Fig. 6). In *So. parsonsi*, the symphysis is much longer and contributes for all its length to the triturating surface. A large, laterally projecting dentary tubercle is present in *So. brachyrhyncha* (Fig. 6), but totally absent in *So. parsonsi*. As a result of the aforementioned features, the outline of the triturating surface is much different in the two species. In *So. brachyrhyncha*, the triturating surface is short and wide, with concave lateral margins (Fig. 6). The two rami of the triturating surface widen posteriorly and are partly separated anteriorly by a medial trough at the symphysis. The triturating surface of *So. parsonsi* is elongated and V-shaped, with straight lateral margins. The two branches of the triturating surface are wider anteriorly at the level of the symphysis and decrease in width posteriorly in *So. parsonsi*. The coronoid process of *So. brachyrhyncha* is stouter and shorter, and has a striated upper part (Fig. 6). In *So. parsonsi*, the coronoid process is higher and smoother. The anterodorsal process of the surangular is more developed in *So. brachyrhyncha* than in *So. parsonsi*, so that only the coronoid process is visible in lateral view in the former species while the posterolateral part of the coronoid main body is exposed laterally in the latter species (Fig. 6). A horizontal ridge is present posteriorly on the lateral surface of the surangular in *So. parsonsi*, but this ridge is apparently absent in *So. brachyrhyncha* (Fig. 6). The splenial is positioned more anteriorly along the mandibular ramus in *So. brachyrhyncha*, reaching posteriorly only to the level of the anteriormost part of the fossa meckelii. As a result, the foramen intermandibularis medius is also located more anteriorly and formed only by the dentary and splenial. In *So. parsonsi*, the splenial reaches posteriorly to the level of the middle of the fossa meckelii and the foramen intermandibularis medius is formed by the dentary, the splenial and the coronoid. Finally, the foramen intermandibularis caudalis is located more anteriorly (level with the anterior part of the fossa meckelii) in *So. brachyrhyncha* (Fig. 6). This foramen is located more posteriorly (level with the posterior part of the fossa meckelii) and reduced in size in *So. parsonsi*.

Our knowledge of the shell morphology of *So. brachyrhyncha* is limited by the fact that only some elements of the shell can be safely assigned to this species and that these elements were found disarticulated and mixed in the assemblage MJSN BAN001-2. We can therefore only provide an incomplete and tentative reconstruction of the carapace and plastron of this new species (Fig. 11). Among similarities with *So. parsonsi*, we can mention the presence of reduced costo-peripheral fontanelles, wide vertebral scales, hyoplastra about as wide as long, and a well ossified plastron with central and lateral fontanelles (for a global comparison with other eurysternids, see Anquetin, Püntener & Joyce, 2017; Püntener, Anquetin & Billon-Bruyat, 2020). Two main anatomical characteristics differentiate *So. brachyrhyncha* from *So. parsonsi*: the development of the costo-peripheral fontanelles and the shape of the central plastral fontanelle. The costo-peripheral fontanelles of *So. brachyrhyncha* extend more anteriorly and posteriorly along the costal series (Fig. 11). Costal 1 is not sutured to the peripherals in the smaller specimens referred to *So. brachyrhyncha* (around 150 mm in carapace length; Fig. 8). A partial sutural contact between costal 1 and peripheral 1 is observed in larger specimens (around 250 mm in carapace length). In contrast, costal 1 is fully sutured to peripherals 1 and 2 in *So. parsonsi* (JM SCHA 70; 184 mm in carapace length). This is also the case in a smaller undescribed specimen (MNB R 2441; around 132 mm in carapace length) from the Late Jurassic of Kelheim, Germany. Similarly, none of the known costals 5 referred to *So. brachyrhyncha* show a sutural contact with adjoining peripherals, even in larger specimens (Fig. 10). In contrast, costal 5 is partially sutured to the peripherals in *So. parsonsi* (JM SCHA 70). The condition in the smaller undescribed specimen MNB R 2441 is uncertain, but narrow fontanelles may have been present. Although the development of costo-peripheral fontanelles is often subject to ontogenetic variations (Anquetin & Joyce, 2014; Shao et al., 2017), the above comparisons clearly indicate that shell ossification is more advanced in *So. parsonsi* than in *So. brachyrhyncha*. This can also be observed in the plastron, where the contact between the hyoplastron and peripherals is more ossified in *So. parsonsi* (JM SCHA 70) than in similarly sized specimens of *So. brachyrhyncha* (Fig. 11).

As far as the shell is concerned, the most striking anatomical difference between *So. brachyrhyncha* and *So. parsonsi* is the shape of the central plastral fontanelle. It is somewhat escutcheon shaped and formed mostly by the hypoplastra in *So. brachyrhyncha* (Figs. 11, 13 and 14), whereas it is oval and equally formed by the hyo- and hypoplastra in *So. parsonsi*. Intraspecific variability of the central plastral fontanelle is known in thalassochelydian turtles, for example in *Plesiochelys etalloni* and *Plesiochelys bigleri* (Anquetin, Püntener & Billon-Bruyat, 2014; Püntener, Anquetin & Billon-Bruyat, 2017a, 2017b). However, the variation mainly concerns the size and absence/presence of the fontanelle, but never such a difference in shape as the one observed between *So. brachyrhyncha* and *So. parsonsi*. Moreover, the particular shape of the central plastral fontanelle in *So. brachyrhyncha* can be repeatedly observed in several isolated hyo- and hypoplastra.

### *Tropidemys*? *langii*?

We herein assign two hypoplastra (MJSN BAN001-2.29) to a juvenile plesiochelyid turtle (Fig. 16). However, what stands out with the hypoplastra MJSN BAN001-2.29 is their relatively wide plastral bridge. Measured from the deepest point of the inguinal notch, the bridge part of these hypoplastra is about as wide as long. This is also a characteristic feature of adult specimens of the plesiochelyid *Tropidemys langii* (e.g., MJSN VTT006-563 and MJSN VTT006-52; Püntener et al., 2014). Unfortunately, juvenile plastra of *T. langii* have not yet been described. In contrast, the bridge part of the hypoplastron in *Plesiochelys etalloni* and *Plesiochelys bigleri* is always longer than wide (Püntener, Anquetin & Billon-Bruyat, 2017a). This is especially pronounced in adult specimens, where the bridge part of the hypoplastron is often about twice as long as wide. This part of the hypoplastron is also clearly longer than wide in juvenile specimens of *P. etalloni* (NMS 9148) and *P. bigleri* (MJSN SCR010-327), although less pronounced than in the adults. In *Craspedochelys jaccardi*, a plesiochelyid with a wide shell, the bridge part of the hypoplastron is relatively wider than in the two aforementioned *Plesiochelys* species, but it is still longer than wide in both adults (e.g., MHNN FOS 977 and NMS 9174) and juveniles (MG-LNEG 28).

Hence, the hypoplastra of MJSN BAN001-2.29 most likely belong to a juvenile specimen of *T. langii*. This attribution is, however, only tentative, as juvenile plastra of this species have not yet been described. *Tropidemys langii* is by far the most abundant turtle in the Banné Marls (Püntener et al., 2014), which somehow supports our tentative assignment.

### Number of specimens, ontogeny, and taphonomical considerations

The assemblage MJSN BAN001-2 is composed of more than 180 turtle shell bones and a turtle cranium (see Material). Most of this assemblage probably represents the new species *Solnhofia brachyrhyncha*, although only the best preserved shell material (only 27 elements) is herein referred to this taxon. Among the bones referred to *So. brachyrhyncha*, the most common are the costals 1 (two left, four right; Fig. 8), the costals 5 (three left, three right; Fig. 10), and the hypoplastra (five left, two right; Fig. 13). This indicates that at least five individuals of this species are represented in the assemblage. The presence of a pair of hypoplastra referable to a plesiochelyid (*Tropidemys*? *langii*?) indicates that at least one specimen of another species is also present in the assemblage. Therefore, the turtle assemblage MJSN BAN001-2 is composed of at least six individuals, but possibly much more.

The pair of hypoplastra referred to a plesiochelyid turtle (MJSN BAN001-2.29) clearly belonged to a specimen in an early ontogenetic stage. Its carapace is estimated to have been about 250 mm in length, while adult specimens usually reach about 450 mm (e.g., *Plesiochelys bigleri*, *Tropidemys langii*; Püntener, Anquetin & Billon-Bruyat, 2017a, 2017b). The estimated carapace length of the specimens referred to *So. brachyrhyncha* varies from 150 to 250 mm, which suggests that different ontogenetic stages are represented in the assemblage. This is corroborated by the ontogenetic variations that we observed in the development of the costo-peripheral fontanelles (see above). It is unknown whether the larger specimens are adults or not. Eurysternids are generally considered as relatively small turtles compared to plesiochelyids (Anquetin, Püntener & Joyce, 2017). However, some taxa are known to reach a carapace length of up to 400 mm (Joyce, 2003; Anquetin & Joyce, 2014) or even more (Püntener, Anquetin & Billon-Bruyat, 2020). The available material of *So. parsonsi* corresponds to relatively small individuals. JM SCHA 70, the only specimen for which the shell is known, has a carapace length of 184 mm and a cranium measuring 59 mm from the tip of the snout to the condylus occipitalis (Joyce, 2000). TM 4023, the holotype and largest specimen known so far, has a cranium measuring 73 mm from the tip of the snout to the condylus occipitalis (Gaffney, 1975), which would correspond to a carapace length of 227.5 mm based on the proportions of JM SCHA 70. If *So. brachyrhyncha* had the same size range as *So. parsonsi* and if what we know of *So. parsonsi* is representative, then the assemblage MJSN BAN001-2 is probably composed of juveniles and adults.

All of the bones of the assemblage MJSN BAN001-2 were found closely together, though completely intermixed and disarticulated. It should be noted that no other vertebrates were represented in the assemblage. Such a selective accumulation of turtles in a closely confined space is exceptional for both the Banné Marls and the Lower *Virgula Marls*. Most other turtle specimens in these levels were discovered clearly isolated from each other (Püntener, Anquetin & Billon-Bruyat, 2017b). Several bone elements show grazing traces of sea urchins on their external surface (e.g., Figs. 13E and 16). Similar traces have also been observed on shell bones of *Plesiochelys etalloni* from the Turtle Limestone of Solothurn (Meyer, 2011) and *Tropidemys langii* from the Banné Marls of Porrentruy (Püntener et al., 2014). Meyer (2011) attributed the traces from Solothurn to the ichnospecies *Gnathichnus pentax* Bromley, 1975, and suggested that they were produced by *Hemicidaris mitra* Agassiz, 1840, a species that is also common in the Banné Marls of Porrentruy (Comment et al., 2015). The presence of such grazing traces indicate that the shells were not immediately buried, as *Hemicidaris mitra* probably fed on a dense algal cover that grew on the shell bones (Meyer, 2011). The borders and surface of several bones show signs of abrasion, suggesting postmortal transportation or reworking of at least a part of the assemblage. A few bones also possess round depressions on the external surface (1–8 mm in diameter), but the origin of these marks is unknown (i.e., crocodylomorph bite marks, skin parasites, etc.). Apart from the above considerations, we have no clue as to the origin of this dense, selective accumulation of turtle bones.

## Institutional Abbreviations

ICHLU: Ichnospace, Luzech, France
JM: Jura-Museum, Eichstätt, Germany
MG-LNEG: Museu Geológico, Laboratório Nacional de Energia e Geologia, Lisbon, Portugal
MHNN: Muséum d’histoire naturelle, Neuchâtel, Switzerland
MJSN: Jurassica Museum (formerly Musée Jurassien des Sciences Naturelles), Porrentruy, Switzerland
MNB: Museum für Naturkunde, Berlin, Germany
MNHN: Muséum National d’Histoire Naturelle, Paris, France
NMS: Naturmuseum Solothurn, Switzerland
TM: Teylers Museum, Haarlem, Netherlands

## Locality Abbreviations

BAN: Le Banné, Porrentuy, Canton of Jura, Switzerland
BEB: Béchat-Bovais, Courtedoux, Canton of Jura, Switzerland
CNJ: Canjuers, Var, France
SCHA: Schamhaupten, Bavaria, Germany
SCR: Sur-Combe-Ronde, Courtedoux, Canton of Jura, Switzerland
VTT: Vâ Tche Tchâ, Courtedoux, Canton of Jura, Switzerland

## Additional Information and Declarations

### Data Availability

The following information was supplied regarding data availability: The specimens described in this work are all stored at the Jurassica Museum (Porrentruy, Switzerland) under the following accession numbers: MJSN BAN001-2.1 to MJSN BAN001-2.29, MJSN SCR010-1214, and MJSN BEB011-13. 3D model of cranium MJSN BAN001-2.1 (*So. brachyrhyncha*) available from MorphoMuseuM. DOI 10.18563/m3.sf.536 3D model of TM 4023 (*So. parsonsi*) is available at MorphoSource: https://www.morphosource.org/Detail/ProjectDetail/Show/project_id/462 - S10665: https://www.morphosource.org/Detail/SpecimenDetail/show/specimen_id/10665

### New Species Registration

The following information was supplied regarding the registration of a newly described species: Publication LSID: urn:lsid:zoobank.org:pub:C999A877-3080-490B-8202-6E98B621366E *Solnhofia brachyrhyncha* n. sp. LSID: urn:lsid:zoobank.org:act:F6C29ABB-1CCC-4E91-8653-95AE1691CF4E.
